# The Interplay of Cross‐Organ Immune Regulation in Inflammation and Cancer

**DOI:** 10.1002/mco2.70249

**Published:** 2025-06-15

**Authors:** Jie Dou, Jinzuo Jiang, Yangtao Xue, Xiaoqi Jiang, Yongzhuo Jiang, Peng Xiao, Junjie Xu

**Affiliations:** ^1^ Department of General Surgery Sir Run‐Run Shaw Hospital Zhejiang University School of Medicine Hangzhou China; ^2^ National Engineering Research Center of Innovation and Application of Minimally Invasive Instruments Hangzhou China; ^3^ Hangzhou Institute for Advanced Study University of Chinese Academy of Sciences Hangzhou China; ^4^ The Second Clinical Medical School Nanjing Medical University Nanjing Jiangsu China; ^5^ Department of Gastroenterology Sir Run‐Run Shaw Hospital Zhejiang University School of Medicine Hangzhou China

**Keywords:** cancer, cross‐organ immune regulation, gut–liver–brain axis, immune crosstalk, inflammation, systemic immunity

## Abstract

Organs dynamically interact with each other through immunomodulation to create a systemic immune response and influence disease progression. While traditional studies have tended to focus on single‐organ immunity, recent studies have placed greater emphasis on reciprocal immune interactions between organs, such as those between the gut, liver, and brain. However, the precise mechanisms underlying these interorgan immune interactions remain unclear. Here, we synthesize the molecular and cellular bases of cross‐organ immune regulation in the context of inflammation and neoplasia. Specifically, we describe the immune coordination between the gut, liver, and brain and how they immunomodulate other organs (including the thyroid, lung, cardiovascular system, kidney, bone, and skin). In addition, we explore clinical therapies that target these cross‐organ immune modulations, the limitations of the treatments, and the potential benefits for patients. We also conclude by highlighting innovative technologies such as multiomics analysis, machine learning, and organ‐on‐a‐chip platforms, which are providing unprecedented insights into interorgan immunity. Elucidating these mechanisms will advance precision medicine and enable the development of targeted therapies for diseases caused by cross‐organ immunity.

## Graphical abstract

Organs interact dynamically through immune regulation, influencing systemic immune responses and disease progression. We review molecular and cellular mechanisms of cross‐organ immune regulation in inflammation and cancer, focusing on gut, liver, and brain interactions with other organs. Advances in multiomics, machine learning, and organ‐on‐a‐chip technologies provide new insights for precision medicine strategies.

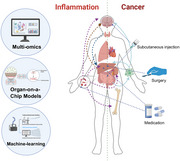

## 1 Introduction

Cross‐organ immunoregulation refers to the process of interaction and regulation of the immune system between different organs with the aim of maintaining the overall immune balance of the body. This regulatory mechanism plays a crucial role in pathological processes such as inflammation and cancer. An in‐depth study of cross‐organ immune regulation can help reveal the pathogenesis of complex diseases. The immune system not only plays a key role in defense against pathogens [1] but also participates extensively in the functional regulation of multiple organ systems. The immune system secretes cytokines [2], regulates the activity of immune cells [3], and mediates inflammatory responses [4] to maintain systemic immune homeostasis.

Previous studies have focused mainly on the immunoregulatory mechanisms of organs themselves, with less attention paid to interorgan immune interactions in the context of inflammation and tumors. Local immune regulation provides key clues but remains insufficient to fully unravel the systemic immune dynamics that drive disease progression. Cross‐organ immunomodulation not only enhances specific organ function, but may also lead to multiorgan damage in pathological states. Therefore, in‐depth analysis of its regulatory mechanisms is important for the development of intervention strategies for multiorgan related diseases.

The focus of this review is on how different organs influence each other through immune signaling, especially under disease conditions such as inflammation and cancer. Sections 2–5 of the article respectively introduce: (1) immune crosstalk between the gut, liver, and brain, and their immune regulation of other organs such as the cardiovascular system and thyroid; (2) clinical approaches, side effects, and impacts of immunotherapy; (3) the role of key technologies such as single‐cell sequencing in studying cross‐organ immune regulation; (4) a summary of the main research findings and proposed future research directions.

The focus of this review is to explain how immune signals mediated by immune cells and metabolites maintain the systemic immune balance, to explore the effects of anti‐inflammatory drugs, metabolic regulators, and immune checkpoint inhibitors (ICIs) on distant organs, to describe the side effects of these treatment methods, and to introduce the application of cutting‐edge technologies such as single‐cell sequencing, spatial transcriptomics, and multiomics analysis in cross‐organ immunology research, highlighting their potential in promoting precision medicine and personalized immunotherapy.

## 2 Interorgan Regulation of the Immune Microenvironment

### 2.1 Gut‐Based Regulation

The microenvironment of the gastrointestinal tract exhibits specificity and functions as a miniature ecosystem within the human body. Microorganisms and their metabolites are present in various locations within the body, including the gut [5, 6], immune cells or tissues [7, 8], and the enteric nervous system (ENS) [9]. The presence of these structures enhances the likelihood of the gut being interconnected with other organs, and maintaining intestinal homeostasis is crucial for human health. The gastrointestinal tract houses the most substantial population of immune cells within the human body, which actively monitor the luminal environment [8], facilitating the physiological communication between the gut and other organs of the body. This crosstalk has the potential to modulate the immune microenvironment, influence the host's immune response, and sustain homeostasis within the body. A deeper understanding of the interactions between various organs will enable us to implement interventions in the gastrointestinal tract to combat diseases and enhance human health [6].

#### 2.1.1 Gut–Liver Axis

The gut–liver axis involves a two‐way interaction between the gut, its microbes, and the liver. This relationship is crucial for maintaining liver health and function. Disruptions in this axis can lead to chronic liver diseases (CLDs). For example, alcohol consumption alters the gut microbiome. These changes promote inflammation, which damages the liver. The gut microbiota also drives the progression of nonalcoholic fatty liver disease (NAFLD). Similarly, it affects autoimmune liver diseases (AILDs). These connections highlight why modifying the gut microbiota matters. Targeting the microbiome may offer strategies for treating liver disorders. Managing gut health could become a key approach in improving liver outcomes.

A bidirectional relationship is established between the gastrointestinal tract and its associated microbiota, as well as the liver, a phenomenon referred to as the gut–liver axis. The gut–liver axis relies on the portal vein to enable direct transport of gut‐derived substances to the liver. This system also involves a liver‐driven feedback loop that regulates bile and antibody delivery back to the digestive tract. Additionally, gut microbes are essential for maintaining the intestinal barrier's integrity and supporting normal liver function. It contributes to nutrient provision, the maturation of the immune system, and the regulation of hepatocyte proliferation and differentiation [10]. Therefore, the regulation of the microbial community is crucial for preserving the homeostasis of the gut–liver axis and facilitating optimal liver function [11]. The impact of gut microbiota is significant and cannot be ignored.

**FIGURE 1:**
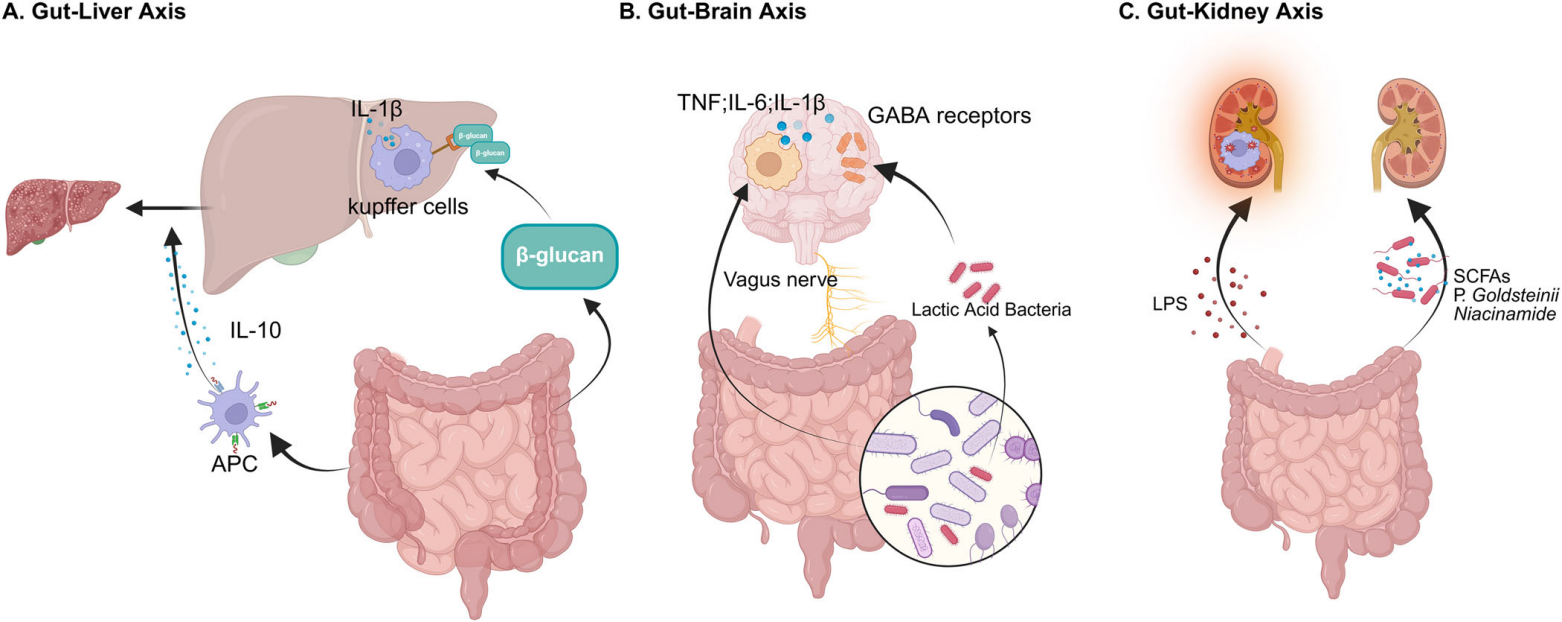
Cross‐organ immune regulation by the gut. (A) Gut–liver axis: microbial adhesion to the intestinal epithelium helps induce IL‐10 through intestinal antigen‐presenting cell subsets, exacerbating liver inflammation and fibrosis; β‐glucan in the gut enters the liver and interacts with the C‐type lectin‐like receptor CLEC7A on Kupffer cells and other bone marrow‐derived cells, increasing the expression and secretion of interleukin‐1β (IL‐1β), thereby triggering liver inflammation. (B) Gut–brain axis: the gut microbiome communicates with the brain through a variety of pathways, including the immune system and vagus nerve. Lactic acid strains can affect the expression of central γ‐aminobutyric acid (GABA) receptors through the vagus nerve; The gut microbiota also promotes the release of a variety of proinflammatory cytokines from brain immune cells, including tumor necrosis factor (TNF), interleukin‐1β (IL‐1β), and interleukin‐6 (IL‐6). (C) Gut–kidney axis: gut microbiota metabolites such as short‐chain fatty acids (SCFAs) and niacinamide, as well as some specific microbiota such as P. Goldsteinii, which plays an important role in the protection of the kidneys; Dysbiosis of the gut microbiosis can exacerbate kidney damage through mechanisms such as lipopolysaccharide (LPS)‐induced oxidative stress.

Kim et al. [12] demonstrated that microbial adhesion to the intestinal epithelium contributes to the regulation of the balance between proinflammatory and anti‐inflammatory T cell responses by inducing IL‐10 through intestinal antigen‐presenting cell subsets. In liver disease, higher serum IL‐10 levels are also associated with higher disease severity [13], Kupffer cells interact with Toll‐like receptor (TLR)2/3 to induce IL‐10 production [14], so endogenous IL‐10 is critical in maintaining immune tolerance, while exogenous IL‐10 administration can exacerbate liver inflammation and fibrosis [15], and studies have shown that IL‐10 production by effector CD8+ T cells promotes their own intrahepatic survival, thereby supporting rather than inhibiting hepatic immunopathology [15] (Figure 1A). It is therefore plausible that the gut may influence liver function within the immune microenvironment through the action of cytokines.

Alcohol consumption is a major driver of CLD by impairing the gut–liver axis through multiple pathways. These include disturbances in the gut microbiome, degradation of the mucus and epithelial barriers, and reduced antimicrobial peptide (AMP) synthesis. Such alterations increase microbial translocation to the liver, promoting inflammation in hepatic tissues. A recent study has shown that prolonged exposure to alcohol results in an increased abundance of fungal communities within the gastrointestinal tract and facilitates the translocation of fungal β‐glucan into the systemic circulation. This β‐glucan is known to provoke liver inflammation by interacting with the C‐type lectin‐like receptor CLEC7A found on Kupffer cells and other bone marrow‐derived cells in the liver. This interaction subsequently elevates the expression and secretion of interleukin‐1 beta (IL‐1β), which ultimately leads to hepatocellular injury and plays a significant role in the pathogenesis of alcohol‐related liver disease [16]. In alcoholic liver disease, the gut microbiota actively shapes the liver's immune environment. This interaction drives disease progression by disrupting immune balance.

Gut microbes play a key role in driving NAFLD. Recent studies show that a “leaky gut” allows molecules like lipopolysaccharides (LPS), free fatty acids (FFA), and bile acids to enter the bloodstream. These substances trigger inflammation by activating proinflammatory signals. This interplay further impacts the development and progression of NAFLD, highlighting the role of the gut–liver axis in this condition [17].

In AILDs, a significant association exists between intestinal inflammation and mucosal immune activation, particularly concerning inflammatory bowel disease (IBD) and primary biliary cholangitis. Intestinal biological disorders can result in immune cell dysfunction and abnormal bile acid signaling [18].

Gut microbes can also influence cancer treatment by regulating the immune system [19]. Through the biliary tract, hepatic portal vein, and biliary secretions, intestinal bacteria and their metabolites can be transported to the liver, potentially inducing liver inflammation and carcinogenesis [20]. There is evidence that the gut microbiota varies significantly between HCC patients and normal people, including differences in species, quantities, and their derivatives [21]. In addition, disturbances in the gut microbiota lead to the secretion of IL‐25, which can subsequently induce CXCL10, promoting the migration of HCC [22]. It is evident that the gut can influence the process of HCC through immunomodulatory mechanisms.

Therefore, the crosstalk between the gut and liver appears to be a potential target for liver disease and modulating the gut microbiota may serve as an effective strategy to control the progression of various liver diseases [23, 24].

#### 2.1.2 Gut–Brain Axis

The gut microbiome is central to the microbiome–gut–brain axis. It shapes brain health and behavior through immune pathways and other mechanisms. Disruptions in gut bacteria can destabilize mood regulation and worsen diseases, underscoring why gut health is vital for overall wellness. Additionally, gut–brain interactions activate the central nervous system (CNS) during intestinal inflammation. This process may fuel psychiatric disorders such as anxiety and depression. These findings highlight the need for deeper research into how gut dysfunction contributes to CNS conditions.

The gut microbiome is instrumental in facilitating the bidirectional communication between the gut and the CNS, a relationship commonly referred to as the microbiome–gut–brain axis. This concept has gained increasing recognition in contemporary research. The microbiota engages in communication with the brain via multiple pathways, which encompass the immune system, tryptophan metabolism, the vagus nerve, and the ENS. This interaction involves microbial metabolites, including short‐chain fatty acids (SCFAs), branched‐chain amino acids, and peptidoglycan [25, 26]. These microbiota metabolites also influence the tumor microenvironment and enhance immunity to tumors, regulating microglia homeostasis, and so on [27, 28]. Gut microbes and their metabolites can play a key role in nutritional interventions to maintain brain health as regulators of blood–brain barrier (BBB) integrity and brain health [29, 30]. The lack of probiotics in the gastrointestinal tract not only adversely affects the gut, but has also been shown to affect the central hypothalamic–pituitary–adrenal (HPA) axis and monoaminergic activity, which are associated with the etiology of depression [31]. After rats were treated with probiotics, the symptoms of “depression” decreased [31]. Gut microbiota and their metabolites significantly contribute to the regulation of mood, and dysbiosis may exacerbate various diseases. In humans, the intestines and lungs are primary targets of the physiological response elicited by ischemic attacks. Mucosal microorganisms significantly contribute to immune regulation and metabolic processes, and they influence the permeability of the BBB. Abnormalities in intestinal microbiota, alterations in the intestinal microenvironment, lung infections, chronic diseases, and the use of mechanical ventilation may exacerbate the outcomes of ischemic stroke [32]. Gut microbiota significantly influence the host's immune response, psychological well‐being, and overall health of the host [33]. In addition, the ENS comprises millions of neurons and glial cells that are embedded within the walls of the gastrointestinal tract, where these neurons and glial cells play a significant role in intestinal immunity. It controls important functions of the gut and, similar to the gut microbiota, has the capacity to interact with the immune system, gut microbiota, and the gut–brain axis [9], all of which play a significant role in gut‐based cross‐organ immune regulation.

The vagus nerve acts as a key pathway for transmitting signals from the gut to the brain. Studies has shown that ingested lactobacillus strains can affect emotional behavior and the expression of central gamma‐aminobutyric acid (GABA) receptors in murine models via the vagus nerve. Importantly, mice that underwent vagus nerve resection exhibited no neurochemical or behavioral changes [34]. In the context of inflammatory conditions, the intestine activates a response in the CNS via the vagus nerve [35, 36]. Gut microbiotas promote the release of several proinflammatory cytokines by immune cells, including tumor necrosis factor (TNF), IL‐1β, and IL‐6 (Figure 1B). This process subsequently influences tight junctions, leading to an increase in barrier permeability [37, 38]. Under conditions of immune dysregulation, increased intestinal permeability may mediate functional alterations in the CNS.

Typical effects of intestinal inflammation on the CNS encompass an exaggerated response of the HPA axis and an imbalance of serotonergic activity [33]. People with comorbidities characterized by persistent intestinal inflammatory features, such as irritable bowel syndrome (IBS) and IBD, are at an increased risk of experiencing anxiety and depression [39, 40]. The impact of intestinal inflammation on the brain is likely mediated by various immune factors. Specific inhibitors or antagonists of cytokines such as cytokines IL‐1β, IL‐6, and TNF can alleviate these CNS changes caused by intestinal inflammation, such as the use of cyclooxygenase inhibitors [41] and disruption of vagus nerve signaling [42].

Investigating the gastrointestinal tract's involvement in CNS disorders may elucidate mechanisms of host‐microbiome crosstalk and drive the development of innovative prognostic tools and therapeutic interventions for CNS pathologies.

#### 2.1.3 Gut–Kidney Axis

Growing evidence highlights the critical contribution of gut microbial communities to renal disorders. As shown in Figure 1C, emerging research suggests that these microbial populations contribute to the development and progression of kidney diseases via diverse pathways, including metabolic regulation and immune modulation. First, gut microbiota metabolites such as SCFAs and niacinamide play an important role in renal protection, and oral probiotics such as Lactobacillus casei can alleviate kidney damage and delay renal function decline by modulating these metabolites [43]. Second, intestinal dysbiosis can exacerbate kidney injury through mechanisms such as LPS‐induced oxidative stress, while antibiotic intervention can significantly improve colitis‐related renal inflammation and injury [44]. In addition, the gut microbiota is closely related to kidney function, and its interaction is influenced by metabolic states such as diabetes mellitus [45], and metabolic imbalances due to dysbiosis can accelerate the progression of chronic kidney disease (CKD) [46].

In specific kidney diseases, the gut microbiota has also shown a unique role. For example, a decrease in butyrate‐producing and oxalate‐degrading bacteria is associated with the development of calcium oxalate nephrolithiasis [47] and may increase the risk of transitional cell carcinoma and renal cell carcinoma [48]. In kidney transplant recipients, dysbiosis of the gut microbiota was significantly associated with quality of life [49], and specific microbiota such as P. Goldsteinii may exert a renal protective effect through metabolic regulation [50]. In addition, the gut microbiota is also involved in regulating the efficacy of drugs, such as berberine in improving CKD by altering microbiota composition and inhibiting uremic toxin production [51].

In general, the gut microbiota is involved in the pathophysiological process of kidney disease through mechanisms such as immune regulation. A better understanding of the mechanism of action of the gut–kidney axis will provide new perspectives and strategies for the prevention, diagnosis, and treatment of kidney diseases.

#### 2.1.4 Other Gut‐Based Axis

In terms of bone metabolism, the gut microbiota is closely related to bone remodeling and the occurrence and development of osteoporosis [52, 53]. Restoring intestinal microbiota balance, transplanting healthy flora, or supplementing with specific cultures may improve bone health [54]. It was found that l‐arginine‐mediated enhancement of bone mechanical adaptation is mainly due to the activation of a positive nitric oxide‐calcium feedback loop in osteocytes, which provides a new antiosteoporotic strategy for maximizing bone benefits caused by mechanical load through the microbiota–metabolic axis [55]. These findings highlight the important role of the gut microbiota in regulating bone metabolism.

In recent years, a growing body of research has revealed a close link between the gut microbiota and a variety of skin diseases. In chronic spontaneous urticaria, changes in the endogenous microbiota may promote mast cell‐driven skin inflammation by decreasing SCFAs levels and increasing LPS levels [56]. Similarly, gut microbial disorders also affect allergic diseases such as atopic dermatitis (AD) through abnormal immune responses. However, some bifidobacterial species and strains, such as *Bifidobacterium longum* CCFM1029, which is able to improve AD symptoms by modulating immune‐microbial interactions. The mechanism may involve upregulating tryptophan metabolism and producing indole‐3‐carboxyaldehyde, thereby activating an aromatic hydrocarbon receptor‐mediated immune response [57]. In the field of cancer, a two‐sample Mendelian randomized analysis using genome‐wide association study (GWAS) data has further highlighted the important role of the gut microbiota in basal cell carcinoma, melanoma skin cancer, and skin tanning susceptibility [58]. In addition, fecal microbiota transplantation (FMT) and anti‐PD‐1 therapy were able to overcome anti‐PD‐1 resistance in the PD‐1 advanced melanoma subgroup by altering the structure of the gut microbiome and reprogramming the tumor microenvironment [59].

Studies in recent years have shown that the gut microbiota is closely related to the pathogenesis and progression of cardiovascular disease (CVD). In post‐ischemia/reperfusion (I/R) injury, the gut microbiota plays a key role in the inflammatory microenvironment. Elimination of intestinal bacterial translocation by using an antibiotic cocktail can alleviate excessive inflammatory response and bone marrow cell mobilization, thereby alleviating myocardial I/R injury [60]. In addition, the gut microbial metabolite phenylacetylgutamine (PAGln) is closely associated with the presence and severity of heart failure (HF), and modulating the gut microbiota, especially the production of PAGln, may be a potential strategy for the treatment of HF [61]. Another study noted that the gut microbial metabolite trimethylamine N‐oxide (TMAO) is also involved in the pathogenesis of CVD [62]. Therefore, gut bacteria and their metabolic pathways are gradually receiving more attention as potential targets for CVD intervention [63].

### 2.2 Liver‐Based Regulation

The liver is a vital organ that regulates intricate networks of mediators and modulates interactions among various organs during inflammatory dysregulation. It plays a central role in protein synthesis [64], the metabolism of toxins and drugs [65], as well as the regulation of immunity and host defense [66, 67].

#### 2.2.1 Liver–Brain Axis

**FIGURE 2:**
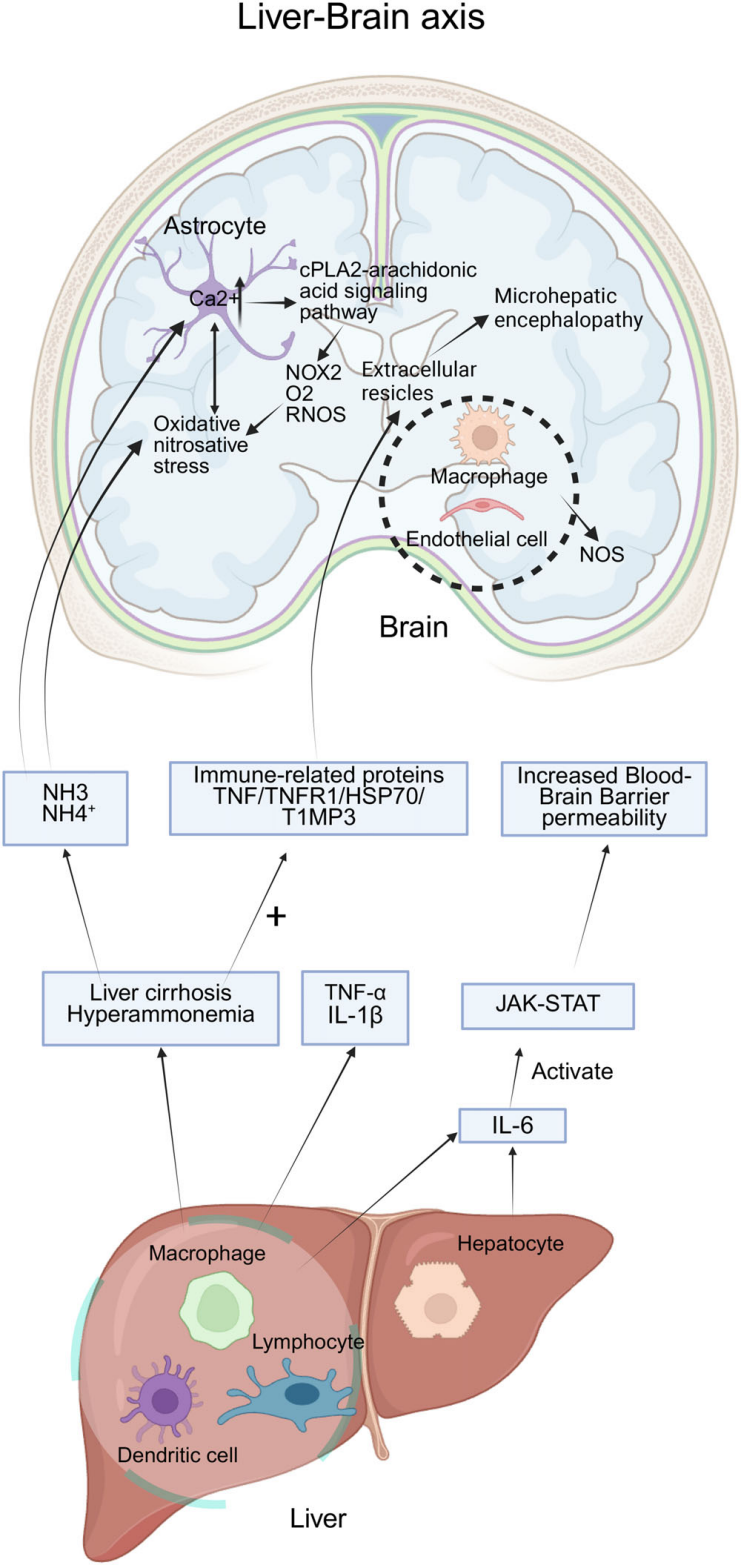
Cross‐organ immune regulation by the liver to brain: liver‐derived inflammatory cytokines (e.g., TNF‐α, IL‐1β, IL‐6) enter the bloodstream and increase blood–brain barrier (BBB) permeability and promoting neuroinflammation. Liver cirrhosis and hyperammonemia upregulate immune‐related proteins (e.g., TNF, TNFR1, HSP70, TIMP3, glutamine synthetase), driving systemic inflammation and altering extracellular vesicles (EVs), which may cross the BBB and contribute to minimal hepatic encephalopathy (MHE). Additionally, hyperammonemia synergizes with inflammation, leading to oxidative/nitrosative stress in astrocytes via an NMDAR‐dependent mechanism, contributing to astrocyte swelling and neuronal dysfunction.

The communication between the liver and the brain is mainly realized through the liver–brain axis, which is an information interaction system based on the circulatory system, vagus nerve [68], immune system [69], neuroendocrine system [70], and contains a variety of neuroactive substances. Here, we focus on the immune‐regulatory mechanism between the liver and the brain. Figure 2 illustrates the immunological signaling pathways through which the liver modulates brain function, thereby contributing to the pathophysiology of hepatic encephalopathy (HE).

As a key immune organ, alterations in the immune status of the liver can affect the systemic immune response, especially the brain [71], and lead to HE. Liver disease may trigger systemic inflammation, where liver immune cells, including macrophages, dendritic cells, and lymphocytes, release proinflammatory factors like TNF‐α and IL‐1β, which prompt brain endothelial cells to release secondary messengers such as prostaglandins and nitric oxide, inducing intracerebral changes [72]. For example, proinflammatory cytokines like TNF‐α and IL‐1β stimulate macrophages and brain endothelial cells to produce inducible nitric oxide synthase (NOS) isoforms through which endothelial and neuronal cells generate nitric oxide via NOS‐mediated oxidation of l‐arginine [73].

Activated hepatocytes and leukocytes generate IL‐6 that binds IL‐6R to activate Janus kinase (JAK)–signal transducer and activator of transcription (STAT) signaling and trigger proinflammatory responses. These inflammatory factors reach the brain through the bloodstream, increase brain endothelial cell permeability, permit the entry of inflammatory molecules, and induce neuroinflammation [74, 75]. Kupffer cells can produce and release cytokines such as TNF‐α, ILs (IL‐1β, IL‐6), and other cytokines. These cytokines can reach the brain through the blood circulation, and at the same time, proinflammatory cytokines alter the permeability of the BBB [76, 77]. This leads to the entry of toxins such as ammonia, triggering a proinflammatory response in the brain [78].

In the pathogenesis of HE, cirrhosis and hyperammonemia cause an increase in the expression of several immune‐related proteins [79] such as TNF, TNFR1, HSP70, TIMP3, and glutamine synthetase [80]. These changes affect the composition of extracellular vesicles (EVs) that can enter the brain, and the compositional changes may be involved in the occurrence of minimal HE.

In addition, ammonia is able to cross BBB in both protonated (NH₄⁺) and deprotonated (NH₃) forms [81], hyperammonemia acts synergistically with inflammation to induce oxidative/nitrosative stress and contributes to elevated intracellular Ca^2^⁺ concentrations in astrocytes through an NMDAR‐dependent mechanism [82]. Ca^2^⁺ overload can be amplified by the cPLA2‐arachidonic acid signaling pathway [83], which further activates NOX2, promotes superoxide anion (O₂−) and RONS production [84], exacerbates oxidative/nitrative stress, and reciprocally promotes astrocyte swelling [85, 86]. HE reflects the clinical outcome of combined pathogenic processes where astrocyte osmotic stress interacts with oxidative and nitrosative stress. The liver drives neuroinflammation and brain dysfunction via inflammatory mediators, EVs, and metabolite‐mediated immune signaling, forming the central pathological mechanism of HE.

Furthermore, the liver is the body's primary metabolic organ, and its metabolites, such as bile acids, can influence the composition and function of gut microbes, which in turn affects the brain via the gut–brain axis [87]. The liver is involved in the process of amyloid‐beta (Aβ) metabolism [88], and imbalances in Aβ have been linked to neurodegenerative disorders, such as Alzheimer's disease (AD) [89]. Changes in liver functioning may influence brain health by affecting the rate of clearance of Aβ.

Consequently, through the production of cytokines and regulating immune cell activity, the liver affects the immune environment of the brain and promotes neuroinflammation, and studying the effects of the liver on the brain can help us better combat brain diseases.

#### 2.2.2 Liver–Thyroid Gland Axis

The thyroid and liver are closely related as the liver regulates thyroid hormone activity through activation, degradation, transport, and metabolic processes.

**FIGURE 3:**
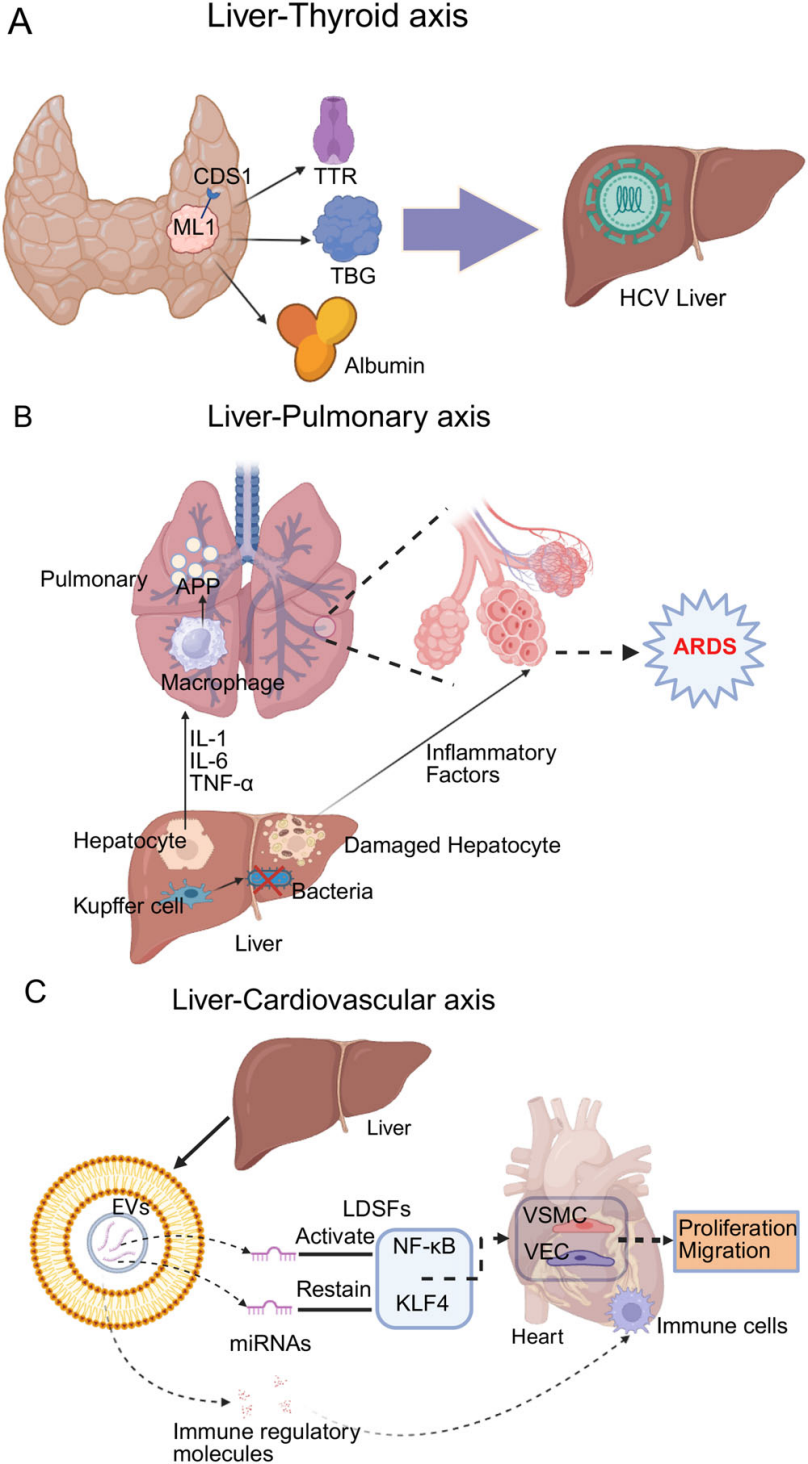
Cross‐organ immune regulation by the liver to other organs. (A) Liver–thyroid axis: the liver plays a role in the activation, inactivation, transport, and metabolism of thyroid hormones, impacting thyroid function. Chronic HCV infection can directly affect thyroid cell function and may trigger autoimmune thyroid disease. (B) Liver–lung axis: illustrating the interaction between the liver and lungs. Activated Kupffer cells release inflammatory factors (IL‐1, IL‐6, TNF‐α), triggering pulmonary macrophages to produce APP. This process may lead to inflammation, increased capillary permeability, alveolar damage, and potentially ARDS. (C) Liver–cardiovascular axis: secretion factors from the liver, including albumin and thyroid hormone‐binding proteins, influence cardiovascular inflammation and function. LDSFs can promote the migration and proliferation of vascular smooth muscle cells (VSMCs) and endothelial cells (VECs), involving KLF4 and NF‐κB pathways.

Liver deiodinases metabolize thyroid hormones altering T4 and T3 plasma concentrations. As an essential component of thyroid hormone metabolism, the liver converts T4 to T3 or rT3, modifying hormone activity and blood levels [90]. Furthermore, the liver synthesizes the primary transport proteins for thyroid hormones, including thyroxine‐binding globulin, transthyretin, and albumin, which facilitate a rapid exchange of circulating thyroid hormones (Figure 3A). As a result, any impairment in liver function may lead to significant changes in the bioavailability of thyroid hormones.

Shen et al. [91] conducted a meta‐analysis of 12 studies and found that the prevalence of antithyroid antibodies was nearly twofold higher and the prevalence of hypothyroidism was more than three‐fold higher in patients with chronic hepatitis C virus (HCV) infection compared with controls.

Studies show HCV may disrupt thyroid function by directly acting on the human ML1 thyroid cell line in vitro; this cell line expresses CD81 on its surface, a receptor essential for HCV entry [92, 93]. Lymphocyte infections, viral proteins, chromosomal irregularities, cytokines including IL‐8, and microRNA molecules are thought to connect HCV infection with thyroid disease [94]. Liver diseases including chronic hepatitis C, cirrhosis, hepatocellular carcinoma (HCC), and cholangiocarcinoma affect thyroid function by changing thyroid hormone levels through altered production of binding proteins, modified deiodinase activity, and decreased liver clearance of reverse T3; these changes impact metabolic and energy balance, highlighting the importance of careful thyroid function monitoring in liver disease patients [95].

#### 2.2.3 Liver–Lung Axis

After hepatocellular injury, the liver may exhibit a reduction in its clearance capabilities, an elevation in the production of deleterious substances, and a disruption of the immune response. These alterations can result in systemic complications, which may include coagulation disorders, an augmented susceptibility to infections, hypoglycemia, an intensified inflammatory response, encephalopathy, and damage to other extra‐hepatic organs, such as the lungs [96]. Hepatic dysfunction is recognized as a key clinical factor influencing the onset, severity, and progression of acute respiratory distress syndrome (ARDS) in critically ill patients. It has also been associated with increased mortality in this population [72, 97, 98]. Figure 3B clearly illustrates how the liver influences the lungs through immune mediators. ARDS is a severe respiratory failure caused by noncardiogenic pulmonary edema, with a hospitalization mortality rate of 35–46% [90, 99, 100]. In patients diagnosed with ARDS, hepatic dysfunction constitutes a key determinant of mortality [101]. Liver injury activates and enhances inflammation in the pulmonary vascular compartments and lower respiratory tracts, leading to important structural and/or functional changes in the lungs, whereas normal liver function exerts a lung‐protective effect and is necessary for recovery from lung injury [102, 103].

The mononuclear phagocyte system of the liver, particularly the Kupffer cells, plays a crucial role in the clearance of bacteria and their byproducts, as well as in mitigating the activation of pulmonary and systemic inflammatory responses [104]. Inflammatory mediators synthesized by the liver, such as IL‐1, IL‐6, and TNF‐α, activate alveolar macrophages [105], thereby increasing the inflammatory response in the lungs, and the liver is responsible for the synthesis of acute‐phase proteins (APPs) [106], which play a crucial role in regulating the systemic and pulmonary inflammatory response, as well as intermediary metabolism, and contribute to the restoration of homeostasis after tissue injury.

#### 2.2.4 Liver–Cardiovascular Axis

CVDs represent the leading cause of morbidity and mortality worldwide [107], and liver immunity and metabolism also have a significant impact on the cardiovascular system [108, 109]. Figure 3C depicts the influence of the liver on the cardiovascular system via immune mediators. In recent years, a growing body of evidence has indicated that liver‐derived secretory factors (LDSFs) play a significant role in the pathogenesis of CVDs. LDSFs refer to a group of substances primarily produced and secreted by the liver, including microbial metabolites, EVs, proteins, and microRNAs. These factors mainly act on various cell types, such as vascular endothelial cells, vascular smooth muscle cells, cardiomyocytes, fibroblasts, macrophages, and platelets. LDSFs are critically involved in regulating multiple physiological processes, including endothelial NOS/nitric oxide signaling, endothelial function, energy metabolism, inflammation, oxidative stress, and vascular calcification [110]. LDSFs have multiple cardiovascular effects that can be beneficial or harmful based on the factor and disease; some LDSFs harm the cardiovascular system. EVs, for instance, carry miRNAs like miR‐1 that inhibit KLF4 expression and activate NF‐κB signaling [111], promoting vascular smooth muscle cell proliferation/migration and causing vascular inflammation/atherosclerosis. These EVs also enhance vascular endothelial cell inflammation and increase microvascular permeability [112]. In addition, exosome miR‐122 secreted by the liver is involved in metabolic cardiomyopathy by inhibiting Arl‐2 and affecting the function of cardiac mitochondria [113], and EVs also carry immune regulatory molecules, which are involved in the recruitment and activation of immune cells. In addition, TMAO [114, 115, 116, 117, 118, 119], fetuin‐B [120, 121], and FXII [122] have been demonstrated to exert detrimental effects on the cardiovascular system.

### 2.3 Brain‐Based Regulation

The interactions among diverse organs within the immune microenvironment, especially those related to the brain, are similar to those witnessed in the liver and gastrointestinal tract. The connection between the nervous system and the immune system is particularly profound, as the CNS not only produces and utilizes immune factors but also has a vital role in the regulation of immune functions [123]. The exploration of cross‐organ interactions within the brain‐centered axis is essential for promoting our understanding of the relationship between the nervous system and the immune system.

#### 2.3.1 Brain–Spleen Axis

**FIGURE 4:**
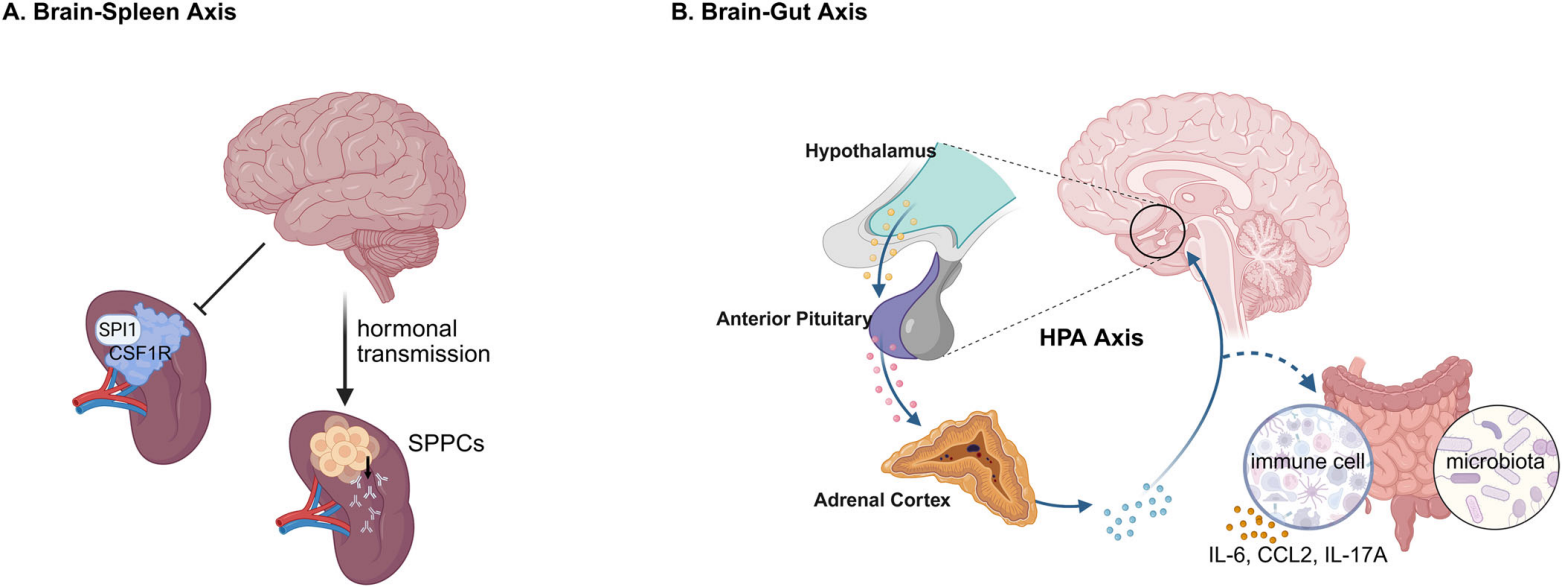
Cross‐organ immune regulation by the brain. (A) Brain–spleen axis: the brain directly interacts with the splenic nerve through hormone transmission, thereby participating in T cell‐dependent splenic nerve regulation, regulating the production of antibodies by splenic plasmacytes (SPPCs); In addition, dysfunction within the brain may affect the interaction of colony‐stimulating factor 1 receptor (CSF1R) with transcription factor PU.1 (SPI1) in the spleen through the brain–spleen axis. (B) Brain–gut axis: the brain influences the composition of the gut microbiota through the hypothalamic–pituitary–adrenal axis, while regulating intestinal immune cell activity. For example, under stressful conditions, the production of interleukin‐17A (IL‐17A), L‐6, and chemokine (C‐C motif) ligand 2 (CCL2) in the gut increases, leading to intestinal inflammation.

The spleen acts as a crucial location for the initiation of the adaptive immune response. In the spleen, numerous immune processes occur, such as antigen presentation, T cell activation, and the differentiation of B cells into antibody‐producing splenic plasmacytes (SPPCs). The splenic nerve governs spleen immunity and is functionally linked to neurons that generate corticotropin‐releasing hormone (CRH), which originate from two specific forebrain regions, namely, the central amygdala and the paraventricular nucleus of the hypothalamus and are implicated in the body's response to stress and threat. Zhang et al. [124] carried out surgical removal of the splenic nerve in mice, and the results showed that the abundance of SPPC was notably reduced after immunization in the denervated mice. On the contrary, the activation of neurons led to an increase in the production of SPPCs [124] (Figure 4A). Evidence exists suggesting that the brain conveys information to the splenic nerve through the vagus nerve, eventually reaching the terminal organ and enabling communication with the spleen [125]. It is highly probable that the brain directly interacts with the splenic nerve through hormonal transmission, thus taking part in the T cell‐dependent regulation of the splenic nerve in the modulation of antibody production.

The colony‐stimulating factor 1 receptor (CSF1R) plays an essential role in regulating the proliferation, differentiation, and survival of microglia and macrophages. Additionally, it has the capacity to interact with the transcription factor PU.1 (SPI1). In a study carried out by Zhang et al., [126] the protein expression levels of CSF1R and SPI1 were evaluated in samples from patients diagnosed with major psychiatric disorders such as major depressive disorder (MDD), schizophrenia, and bipolar disorder. The results disclosed abnormal expression patterns of CSF1R and SPI1 in the spleens of individuals with these psychiatric conditions. This indicates that dysfunction in the brain might influence splenic function through the brain–spleen axis (Figure 4A).

In the effect of the brain on the spleen, brain/nervous system activity may directly control the adaptive immune response of lymphoid organs such as the spleen, thereby giving immune system feedback and regulating immune system function, which plays an important role in the immune microenvironment.

#### 2.3.2 Brain–Gut Axis

The brain–gut crosstalk, apart from the aforesaid intestinal impact on the brain, the gut–brain crosstalk is also significant in regulating various functions of the human body. The subsequent discourse will focus on the axis governing the connection between the intestine and the brain (Figure 4B).

In the efferent pathway of the microbiome–gut–brain axis, the HPA axis affects the composition of the gut microbiota and the body's immune function. It coordinates the release of glucocorticoids by the adrenal glands to restore homeostasis or accelerate intestinal dysfunction through regulating the activity of intestinal immune cells, intestinal function, and others [26]. Stress‐induced dyspepsia has the potential to result in intestinal inflammation via the TH17 cell‐mediated release of IL‐17A [127]. A considerable amount of research has demonstrated that stress can influence the stability of the microbiota, possibly leading to bacterial translocation. In a study on adult mice exposed to a social stressor called social disruption, which raises circulating cytokine levels and enhances the reactivity of the innate immune system, the results imply that chronic stress is related to a decrease in the relative abundance of specific bacterial species and an increase in clostridial species within the cecum. Moreover, this exposure is connected to the activation of the immune system, as shown by the increased production of IL‐6 and chemokine (C‐C motif) ligand 2 (CCL2) [128]. The levels of neuropeptide neurotensin (NT) and CRH found in the brain and intestine are elevated in the serum of children with autism. Additionally, NT was significantly correlated with the existence of gastrointestinal symptoms [129]. In stressful conditions, the brain has the capacity to modify intestinal motor function through the activation of corticotropin‐releasing factor receptors [130]. This evidence indicates that inflammatory processes or pathological circumstances taking place within the brain might have an influence on gastrointestinal function and immune responses.

The cholinergic anti‐inflammatory pathway (CAIP) has been recognized as a significant mechanism through which the brain governs the immune response of the spleen via the vagus nerve. The α7 nicotinic acetylcholine receptor (α7nAChR) is a notable subtype of acetylcholine receptor within the CNS, having a crucial role in neurotransmitter release and the inflammatory response, and functioning as a key regulator of the CAIP. Matteoli et al. [131] showed that the vagus nerve interacts with cholinergic muscle neurons close to muscle macrophages. Additionally, it was found that the activation of α7nAChR modulates ATP‐induced Ca^2+^ responses in resident macrophages of the small intestine [131]. The brain possesses the ability to regulate the resident macrophages within the intestinal muscles expressing α7nAChR through the vagus nerve, thereby subsequently affecting the functionality of the intestinal muscles.

Furthermore, a variety of immune and nonimmune cells exist in the brain/CNS, including the main resident immune cells, microglia, astrocytes, leukocytes, and so on. These cells form a complex interaction network that is capable of modulating the inflammatory response of the gastrointestinal tract [132] and may lead to intestinal immune cell activation.

#### 2.3.3 Other Brain‐Based Axis

The brain and other organs communicate through various means, such as brain‐derived circulating factors entering the periphery via the meningeal‐lymphatic system and outgoing vagus nerve signals, as well as indirect interactions mediated by the HPA axis [132]. The brain utilizes the hypothalamus, a core regulatory structure, to exert its influence on peripheral organs and tissues (such as lungs and pancreas) through sympathetic and parasympathetic nerves. This procedure involves the regulation of immune responses and the preservation of homeostasis, among other functions. Recent experimental investigations have shown that isolated acute brain injury (ABI) can lead to considerable dysfunction of peripheral extracranial organs and systems [133]. Moreover, it has been noticed that a sympathetic rush, which takes place in reaction to a sudden increase in intracranial pressure, causes a temporary increase in intravascular pressure, eventually leading to the deterioration of the alveolar‐caudal membrane [134]. The brain and nervous system have a vital part to play in inflammatory circumstances, which can exert a significant influence on peripheral organs, especially the lungs, in ABI situations.

The hypothalamus acts as a central regulatory center within the cerebral cortex, supervising visceral activities and serving as the regulatory core for both sympathetic and parasympathetic efferent fibers. These efferent innervations have a vital role in regulating the functions of peripheral organs and tissues, such as the pancreatic islets, adipose tissue, and liver [135]. This regulation promotes the cerebral innervation and supervision of these peripheral organs and tissues. The upregulation of bone marrow production triggered by the sympathetic nervous system mediates the proinflammatory element of leukocyte CTRA (conserved transcriptional response to adversity) dynamics and might contribute to a heightened risk of inflammation‐related diseases associated with adverse social circumstances. In reaction to demanding living conditions, mammalian immune cells exhibit CTRA, marked by an upregulation of proinflammatory gene expression. The study conducted by Powell et al. [136] revealed an enhanced myelogenesis output of Ly‐6c (high) monocytes and Ly‐6c (intermediate) granulocytes in mice that experienced repeated social defeats, and these effects were hindered by pharmacological antagonists of β‐adrenergic receptors and the myelogenesis growth factor GM‐CSF. The parasympathetic nervous system has an impact on insulin secretion from the pancreas by releasing acetylcholine. Insulin secretion is regulated by various hormones and neurotransmitters, among which acetylcholine, the main neurotransmitter of the peripheral parasympathetic nervous system, has a considerable role. Gautam et al. [137] showed that the M3 muscarinic acetylcholine receptor in pancreatic β cells is essential for the control of insulin release and the maintenance of glucose homeostasis.

In cases of inflammation or tumors, the brain and CNS can have an impact on other organs through the innervation of sympathetic and parasympathetic nerves. The neurotransmitters released by the brain can promote tumor progression by altering the tumor microenvironment. This phenomenon is governed by a balance defined by the principles of yin and yang [138]. Adrenergic and cholinergic nerves promote the advancement of prostate tumors [139], innervation also leads to the formation of gastric tumors through M3 receptor‐mediated Wnt signaling in stem cells [140], and the neurotransmitter dopamine secretion is locally increased in HCCs, and promotes HCC cell proliferation and metastasis [141]. As a result, neurological interventions could act as an efficient way for handling disease treatment.

## 3 Drugs, Side Effects, Revelations in Cross‐Organ Therapy

### 3.1 Drugs Have an Immunizing Effect on Other Organs by Acting on the Gut

**FIGURE 5:**
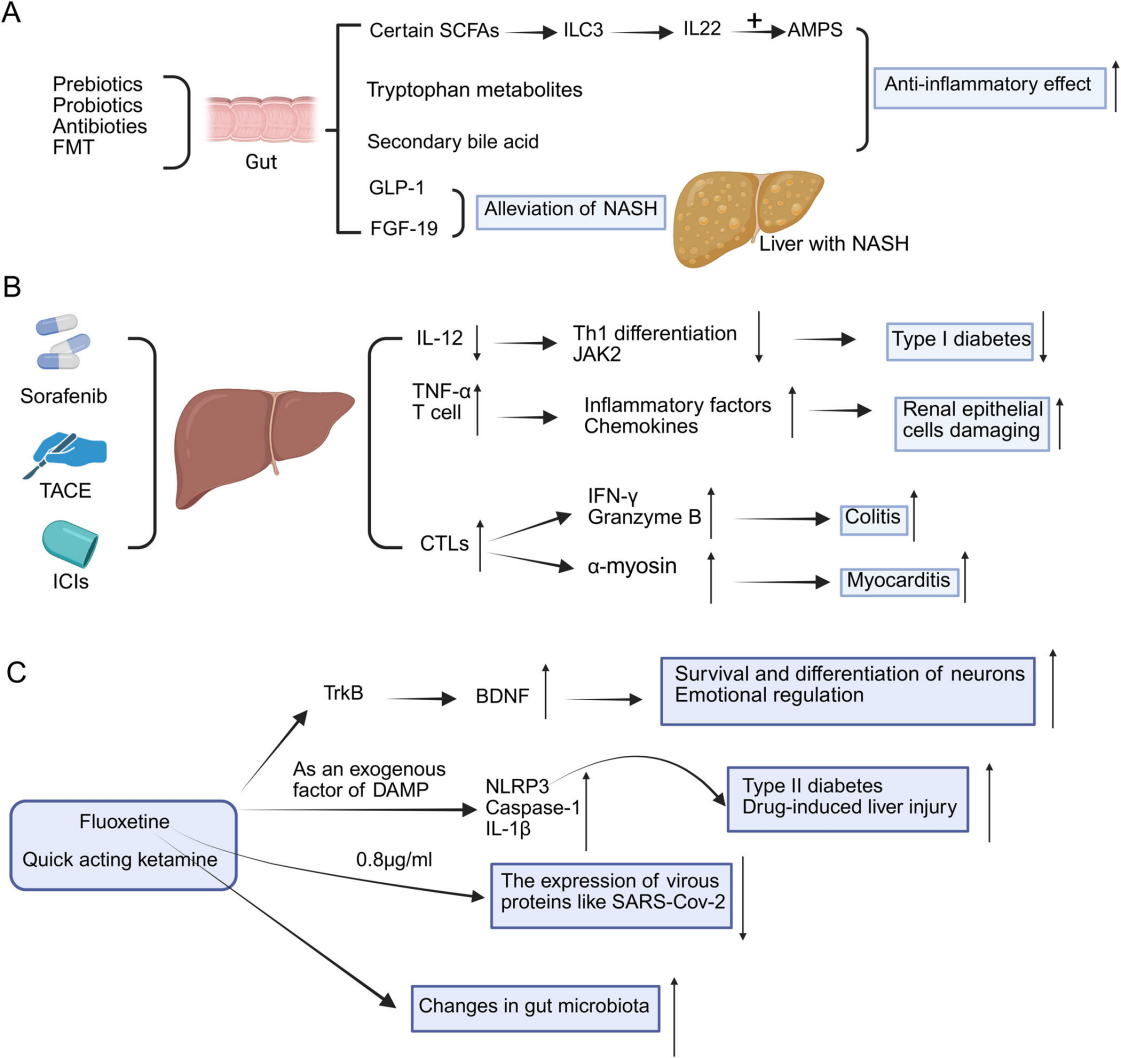
Drugs act on the gut, liver, and brain to affect other organs. (A) Drugs act on the gut to affect other organs: Gut microbiota and its metabolites regulate liver inflammation in NASH. Prebiotics, probiotics, antibiotics, and fecal microbiota transplantation (FMT) modulate gut microbiota composition, influencing the production of various metabolites such as short‐chain fatty acids (SCFAs), tryptophan metabolites, and secondary bile acids. Certain SCFAs promote type 3 innate lymphoid cells (ILC3) to produce IL‐22, which induces AMPs, contributing to an anti‐inflammatory effect. Additionally, GLP‐1 and FGF‐19 alleviate NASH. (B) Drugs act on the liver to affect other organs: Previous clinical reports have shown that sorafenib can significantly lower blood glucose levels in patients with diabetic hepatocellular carcinoma. Sorafenib effectively blocks IL‐12‐induced Th1 cell differentiation and indirectly inhibits JAK2 activation in a dose‐dependent manner in the treatment of hepatocellular carcinoma in NOD mice with type 1 diabetes mellitus, resulting in a reduction in the accumulation of Th1 cells and the expression of pancreatic inflammatory cytokines. During hepatocellular carcinoma treatment, the cytotoxic CD8 t producing IFN‐γ and granzyme B activation of cell populations can make patients more susceptible to colitis. α‐Myosin is also a direct target of cytotoxic CD8 T cells, which act on the heart through the patient's peripheral blood to cause myocarditis. (C) Drugs act on the brain to affect other organs: Antidepressants such as fluoxetine and ketamine enhance BDNF signaling by binding to the TrkB receptor. Fluoxetine can also activate the NLRP3 inflammasome, leading to caspase‐1 activation, IL‐1β secretion, and pyroptosis, which may contribute to diseases like type 2 diabetes and drug‐induced liver injury. Additionally, fluoxetine has been shown to inhibit SARS‐CoV‐2 replication at low concentrations.

The gut microbiota is central to the regulation of the systemic immune response, especially when the gut is subjected to pharmacological treatments. Recent studies have shown that drugs targeting the gastrointestinal tract may alter the integrity of the intestinal barrier, microbial composition, and metabolite production, which can have a profound impact on immune function in distant organs by remodeling immune signaling pathways, and that continued research into these mechanisms may result in new strategies for modulating the immune response in various organ systems. In Figure 5A, the diagram illustrates how the specific drug acts on the intestine, thereby exerting downstream effects on the liver.

#### 3.1.1 Clinically Currently Known Drugs

##### 3.1.1.1 Microbiota Modulators

Gut flora and systemic immune responses are tightly linked [142]. The mechanisms through which the microbiota regulates the immune response primarily involve the production of metabolites and activation of pattern recognition receptors. After acting in the gastrointestinal tract, pharmaceuticals can influence the immune response of various organs throughout the body by modulating intestinal metabolites [143, 144].

SCFAs, tryptophan metabolites, and bile acid derivatives are metabolites with immunoprotective effects [145, 146]. SCFA induce the production of AMPs and mucus by specialized intestinal epithelial cells, which aid in the maturation and proliferation of colonic regulatory T‐cells, thus reducing local inflammatory responses to the intestinal microbiota [147]. SCFAs are essential for the maintenance of the epithelial barrier and intestinal homeostasis, and they also stimulate cell proliferation and differentiation to aid in gut repair [148]. Acetate and propionate activate the Ffar2 receptor, which drives the expansion of CCR6⁺ ILC3s and raises IL‐22 production. IL‐22 boosts antimicrobial activity in epithelial cells by increasing proteins like Reg3α, Reg3β, and Reg3γ that support mucus formation and defense. This response reinforces gut barrier strength. The Ffar2 signaling pathway involving the AKT–STAT3 axis maintains IL‐22 levels and helps ILC3s survive in the gut [149].

In addition, intestinal epithelial cells secrete AMPs [150, 151] which are innate immune effector molecules exhibiting bactericidal, anti‐inflammatory, and antiendotoxin properties [152]. Limiting pathogen–epithelial interactions, their expression may be downregulated by specific pathogens. This downregulation is closely associated with the development of innate immune responses [145, 153].

Drugs targeting the gut microbiota have significant hepatic therapeutic effects; intervening in the gut signaling pathway significantly contributes to the alleviation of hepatic inflammation due to the anatomical and functional specificities of the “gut–liver axis” [11]. Studies have shown that the gut–liver axis is the key to the transfer of inflammatory mediators—including fatty acids, carbohydrates, amino acids, trimethylamine, ethanol, and other bacterial metabolites, as well as bacterial antigens such as LPS—which are critical for transporting these substances to the liver via the portal vein, primarily affecting liver‐resident macrophages: the Kupffer cell [154, 155]. Conversely, the gut affects hepatic function by generating diverse mediators; it modulates immune responses through anti‐inflammatory factors that benefit liver function. These include gut‐derived hormones like glucagon‐like peptide‐1 (GLP‐1), fibroblast growth factor 19 (FGF‐19), and its murine equivalent FGF15, alongside microbiota‐produced secondary bile acids and anti‐inflammatory metabolites such as tryptophan‐derived indoles [154, 156]. Strategies using prebiotics/probiotics/antibiotics or fecal microbial transplants to correct gut dysbiosis in NAFLD may reduce inflammatory signals, repair intestinal barrier dysfunction (thereby lowering portal vein endotoxin levels), and boost endogenous production of beneficial mediators like FGFs, GLP‐1, and secondary bile acids. While preclinical evidence strongly supports microbiota‐based interventions to diminish NAFLD‐associated hepatic inflammation, clinical trials have yet to establish long‐term anti‐inflammatory or antifibrotic benefits [11]. Cutting‐edge microbiota‐targeted therapies—including engineered bacteria, postbiotics, and phages—offer promising avenues for developing more personalized treatment approaches for NAFLD‐associated inflammation [157].

*Hormone analogs* including GLP‐1 receptor agonists semaglutide and liraglutide, along with GLP‐1/GIP dual agonists such as tirzepatide, are used clinically to manage metabolic diseases. Their effects on liver inflammation and fibrosis are indirect, occurring mainly through reduced energy supply and improved hepatocyte metabolism—changes that also regulate immune cell function. For instance, GLP‐1 receptor agonists modify macrophage phenotype and lower proinflammatory cytokine production, thereby affecting inflammatory and fibrotic processes in NAFLD [158]. Phase II trials [159] show GLP‐1 agonism aids nonalcoholic steatohepatitis (NASH) regression; this mechanism is now being fully investigated in larger studies.

In a similar vein, FGF‐19‐like hormones, including aldafermin, demonstrate both anti‐inflammatory and antifibrotic properties by emulating the function of FGF‐19 and modulating bile acid synthesis, as well as glucose and lipid metabolism [160]. These agents are currently undergoing clinical development for the treatment of NASH.

##### 3.1.1.2 Microbiome‐Centered Therapies

Microbiome‐centered precision therapies, including engineered bacteria, probiotics, and phages [161], may offer a promising approach for the individualized treatment of inflammation associated with NAFLD [157]. Prebiotics are nondigestible elements found in food that exert beneficial effects on the host by selectively enhancing the proliferation and/or activity of specific bacterial populations within the colon [162]. Typical prebiotics include human milk oligosaccharides (HMOs), inulin, oligofructose, and oligogalactose; certain dietary fibers like beta‐glucan, arabinoxylan (AX), pectin, and resistant starch—though not classified as traditional prebiotics—also show prebiotic properties. Both prebiotics and these specific fibers foster beneficial gut bacteria proliferation by serving as fermentation substrates, while suppressing pathogenic microorganism growth through ecological niche exclusion. SCFA modulate immune function and reduce the risk of infectious diseases, and their interactions with epithelial and immune cells contribute to the prevention of infections. For example, β‐glucan and AX trigger the activation of the C‐type lectin receptor Dectin‐1 on intestinal epithelial cells, a mechanism that has been implicated in enhancing the immune response to prevent secondary infections [163, 164, 165]. In addition, HMO, AX, and pectin can bind to TLRs and inhibit excessive TLR signaling in intestinal epithelial cells by inducing a tolerogenic subpopulation, thereby enhancing dendritic cell function—a process that protects the gastrointestinal tract and reduces inflammation following infection [166, 167, 168, 169].

In addition to the individual application of prebiotics and probiotics, there exist nutritional strategies that integrate both components into symbiotic formulations. Symbiotics have shown promise in clinical studies and may represent a viable therapeutic alternative in the future [170].

Furthermore, FMT has been explored as a potential approach for restoring gut microbiota balance, involving the transfer of fecal microbiota from a healthy donor into the gastrointestinal tract of the patient [171], has shown certain efficacy in the treatment of ulcerative colitis, IBS, and HE [172].

#### 3.1.2 Limitations

GLP‐1 receptor agonists, including semaglutide, may pose several risks, including hypoglycemia, gastrointestinal side effects, cardiovascular effects, acute kidney injury, an increased risk of diabetic retinopathy, and allergic or injection site reactions [173]. The use of probiotics, similar to other medications, is associated with the potential for considerable side effects [174]. There have been reports indicating that the availability of probiotics, such as Lactobacillus rhamnosus (LGG), may result in immunocompromise, bacteremia, or sepsis in both children and adults suffering from acute severe colitis [175].

#### 3.1.3 Implications for patients

Nutrition significantly affects the composition of the gut microbiota and the immune system [176, 177]. The Western Diet is characterized by excessive fat intake, which is usually associated with overeating, frequent snacking and a long digestion time after meals [178]. The consequences of a high‐fat diet include dysbiosis of the microbiota, damage to the intestinal barrier, increased intestinal permeability, and the entry of harmful bacterial metabolites into the circulatory system, and it is also associated with an intensified inflammatory response [179]. This study deepens our understanding of the relationship between the gut microbiota and host responses, thereby providing more possibilities for regulating the relationship between the two. Nutritional intervention can be used to maintain intestinal homeostasis and enhance resistance to infection [180]. It must be recognized that various dietary components, including minerals, carbohydrates, vitamins, lipids, and proteins, due to their respective characteristics, directly or indirectly affect the interaction between the host and the pathogen through the microbiome in different ways. Clarifying its mechanism of action will provide new ideas for improving health [181]. Dietary intervention should be regarded as an important strategy for regulating the risk of infectious diseases, preventing the invasion of pathogenic microorganisms, reducing the severity of infections and supporting the treatment of infectious diseases [176]. Increasing the intake of dietary fiber, adopting a diverse diet and taking an appropriate amount of prebiotics, probiotics and omega‐3 fatty acids can promote the balance of intestinal flora, thereby having a positive impact on the immune function of various organs in the human body.

### 3.2 Drugs Have an Immunizing Effect on Other Organs by Acting on the Liver

#### 3.2.1 Clinically Currently Known Drugs

Primary liver cancer is the second leading cause of mortality globally [182]. Despite significant advancements in treatment modalities over the years, the clinical prognosis for this condition remains unsatisfactory [183]. Various pharmacological agents are available for the management of different stages of HCC; however, these treatments may also exert immunological effects on other organs throughout the therapeutic process.

The current first‐line treatment for late‐stage conditions continues to be predominantly characterized using sorafenib [184]. Sorafenib is a tyrosine kinase inhibitor (TKI) that impedes tumor angiogenesis by targeting hepatocyte cytokine receptors, vascular endothelial growth factor receptor 2 (VEGFR2), and platelet‐derived growth factor receptor [185]. In addition, sorafenib inhibits tumor proliferation primarily by targeting RAF‐1, B‐Raf, and the kinase activities within the Ras/Raf/MEK/ERK signaling pathway [186]. Although sorafenib affects not only normal hepatocytes but also other cell types, it may alter the distribution and functional states of peripheral blood B cells, T cells, and NK cells in patients [187]. However, these effects primarily result from the direct action of sorafenib on systemic cells and are largely confined to immune responses, such as skin toxicity [188], rather than immune interactions between organs following its action on the liver. A detailed discussion of these adverse effects will be provided in the corresponding subsection of this section. Furthermore, given that tumor cells exhibit significantly higher proliferative and metabolic activity than normal cells, HCC cells may be more sensitive to sorafenib. Therefore, in the context of primary liver cancer, the effects of sorafenib on other organs can be considered a consequence of its primary action on the liver.

Retrospective analyses have reported a notable reduction in blood glucose levels following sorafenib administration in both diabetic and nondiabetic cancer patients [189]. Previous clinical reports have indicated that sorafenib significantly reduces blood glucose levels in patients with diabetic HCC [190]. Beyond metabolic effects, emerging data suggest that sorafenib may interfere with immune regulation. Specifically, it has been shown to inhibit IL‐12‐mediated Th1 cell differentiation in a dose‐dependent manner and indirectly downregulate JAK2 activation [191] (Figure 5B). Such immunomodulatory actions have been linked to a decrease in Th1 cell accumulation and pancreatic cytokine expression and may partially explain sorafenib's observed ability to prevent or reverse type 1 diabetes in NOD mouse models [192].

Transarterial chemoembolization is recognized as the preferred therapeutic approach for HCC in intermediate to advanced stages [193] (Figure 5B). Cisplatin, recognized as a critical therapeutic agent, exerts its effects through direct interaction with DNA, leading to the induction of apoptosis and inflicting damage on mitochondrial structures [194, 195]. TACE‐induced ischemia leads to hypoxia in hepatocytes and liver tissue [196], causing localized inflammation and promoting TNF‐α [197] and T lymphocyte activation [198]. This cascade ultimately leads to the activation of various inflammatory cells, resulting in renal epithelial cell damage.

Furthermore, ICIs are extensively utilized in the management of HCC [199]. ICIs can reactivate T cell receptor‐mediated cytotoxicity and promote CD8^+^ T cell proliferation by acting on the corresponding ligands and receptors [200], enabling the autoimmune system to recognize and kill tumor cells [201]. However, ICIs produce a variety of inflammatory toxicities collectively known as immune‐related adverse events (irAEs) [202]. During HCC treatment, activated cytotoxic CD8⁺ T cells produce IFN‐γ and granzyme B, which may increase the susceptibility of certain patients to intestinal inflammation, such as colitis [203]. α‐Myosin is also a direct target of cytotoxic CD8+ T cells, which act on the heart through the patient's peripheral bloodstream to cause myocarditis [204]. An increasing body of evidence indicates that B cell‐mediated humoral immunity may contribute to thyroid dysfunction, including thyroiditis, following ICIs therapy [205].

Hepatitis B virus (HBV) infection remains a major global health burden. As of 2019, an estimated 296 million individuals tested positive for hepatitis B surface antigen, corresponding to a global prevalence of approximately 3.5% [206]. Chronic HBV infection can lead to prolonged liver inflammation and alterations in the immune microenvironment, making it a major cause of cirrhosis and HCC [207, 208].

Recent evidence indicates that nucleoside analogs (NAs) serve as the principal therapeutic approach for hepatitis B. These nucleoside (acid) analogs are increasingly recognized as a safer treatment alternative that has the potential to reduce the incidence of severe hepatitis [209]. NAs alleviate the systemic inflammatory response triggered by hepatitis by inhibiting HBV replication and reducing liver inflammation [210]. In the context of hepatitis‐related chronic inflammation, NAs improve platelet activation and thrombosis formation by modulating the P2×7‐mediated NLRP3 inflammasome [211], This provides a protective effect on the vasculature and indirectly improves cardiovascular health [212]. Additionally, with the improvement of liver inflammation, the overactivation of the spleen may be suppressed, thereby reducing the occurrence of secondary immune dysregulation.

Interferon (IFN) remains an important therapeutic option in chronic hepatitis B management [213]. Mechanistically, IFN activates the JAK–STAT signaling pathway, leading to the induction of IFN‐stimulated genes (ISGs) [214], which are essential for antiviral defense and immune regulation. The expression of these ISGs further regulates viral replication and the immune response. However, during treatment, exogenous IFN is metabolized by the liver and recruits inflammatory cells [215] to promote T‐cell activation by enhancing MHC II expression, DC maturation, upregulation of costimulatory molecules, and antigen presentation [216] to stimulate a systemic immune response [216]. These immunostimulatory effects are not confined to the liver: in the kidney, IFN‐induced chemokines such as CXCL9 and CXCL10 can contribute to immune‐mediated nephritis. Moreover, IFN enhances immunosurveillance by increasing the expression of TNF‐related apoptosis‐inducing ligand [217], which may play a role in tumor suppression, particularly in cases where viral hepatitis coexists with colorectal cancer [218]. Interestingly, this antitumor potential may be partly attributed to IFN's inhibitory effects on angiogenesis within the tumor microenvironment.

#### 3.2.2 Limitations

First, TKIs such as sorafenib have been shown to modulate immune responses through multiple mechanisms. One notable effect is the inhibition of VEGFR signaling, which can disrupt regulatory T cell (Treg) function and enhance autoimmune activity. This dysregulation is thought to contribute to common dermatologic toxicities observed in clinical practice, including hand‐foot skin reaction [219]. Additionally, the suppression of B cell function by sorafenib may impair antibody‐mediated immune responses, causing immune‐related hematological abnormalities, such as thrombocytopenia, in some patients [220].

ICIs activate self‐reactive T cells that infiltrate the myocardium leading to myocarditis outbreaks [221]. CTLA‐4 expression in normal pituitary cells may promote the toxicity associated with anti‐CTLA‐4 therapy, leading to pituitary inflammation [222]. ICIs also activate inflammatory factors and the microbiome, resulting in elevated baseline levels of IL‐17, which is strongly associated with poor colitis prognosis [223]. A retrospective analysis of 190 HCC patients treated with pembrolizumab monotherapy [224] showed that 18.4% had elevated transaminases, followed by diarrhea (14.2%), dermatosis (13.2%), and thyroid dysfunction (13.2%).

Second, hepatotoxicity is also inevitable during treatment. Cisplatin, a chemotherapeutic agent against HCC, causes oxidative stress in hepatocytes by increasing the production of reactive oxygen species (ROS), inducing hepatocyte degeneration [225] which leads to severe liver injury. Although cisplatin is primarily eliminated via renal excretion, its accumulation within renal tubular epithelial cells contributes to marked nephrotoxicity [226].

Finally, drug resistance is also a challenge to treatment. ICIs improve the prognosis of a subset of patients with HCC, but eventual drug resistance in 20–30% of patients leads to tumor recurrence and progression [227] that may be related to mutations in key antitumor pathways [228] and the overall microbial community composition of patients [229]. Moreover, the efficacy of IFN‐based therapies may decline over time due to progressive T cell exhaustion, further complicating dosing strategies in clinical settings.

#### 3.2.3 Implications for Patients

Cisplatin increases the production of ROS thus leading to liver injury, and patients can reduce oxidative stress by consuming more antioxidant foods in their daily diet. In addition, a healthy and regular diet is beneficial for the patient's overall microbiota to remain stable, thus improving resistance to ICIs. Oral corticosteroids combined with high‐dose vitamin B6 can be used to treat or prevent hand‐foot syndrome induced by TKIs.

### 3.3 Drugs Have an Immunizing Effect on Other Organs by Acting on the Brain

#### 3.3.1 Currently Clinically Known Drugs

MDD is increasingly recognized as a considerable global public health concern. The World Health Organization predicts that MDD will become the second leading cause of disability worldwide in the present year [230]. Depression is a chronic and recurrent state that might require lifelong treatment via diverse approaches. A considerable portion of people diagnosed with MDD are still undertreated [231]. In Figure 5C, the diagram illustrates how specific drugs—particularly antidepressants—affect other organs through actions on the brain.

Selective serotonin reuptake inhibitors (SSRIs) are a newer type of antidepressant. Doctors have used them since the 1980s after clinical testing. Six SSRIs are widely prescribed: fluoxetine, sertraline, paroxetine, fluvoxamine, citalopram, and escitalopram. These drugs work by blocking serotonin reuptake in the brain. They target specific transporters on nerve cell membranes. This action boosts serotonin levels between cells. SSRIs have little effect on norepinephrine. Their impact on dopamine is even smaller. Though developed decades ago, SSRIs remain a first‐line treatment. They are still the most common medications for depression today [232].

New research reveals how antidepressants affect brain. Both older drugs like fluoxetine and newer fast‐acting antidepressants like ketamine work through similar pathways. These medications activate TrkB receptors in the brain. Brain‐derived neurotrophic factor (BDNF) plays a key role in neural plasticity and mood regulation [233]. The antidepressant fluoxetine, together with other psychotropic drugs showing similar effects, might act as exogenous damage‐associated molecular patterns that directly trigger the activation of the NLRP3 inflammasome. This process is related to the activation of caspase‐1, the secretion of IL‐1β, and the induction of pyroptosis mediated by Gasdermin D [234]. The dysregulation of NLRP3 inflammasome activity leads to uncontrolled inflammation, which is a contributing factor for various diseases, including type 2 diabetes [235] and drug‐induced liver damage [236]. The reduction of IL‐1β in the rat prefrontal cortex induced by fluoxetine is involved in both transcriptional and posttranscriptional regulatory mechanisms. Moreover, it demonstrates that the activation of the microglial NLRP3 inflammasome acts as a mediator of IL‐1β‐associated neuroinflammation within the CNS during chronic stress periods [237]. Ketamine, which is acknowledged as a prophylactic agent for stress‐induced depression‐like behavior, has been demonstrated to suppress the NLRP3 inflammasome‐driven signaling pathway at higher doses [238]. Moreover, the combination of ketamine and other pharmacological substances might alleviate the side effects related to each drug. The neuropsychiatric disorders associated with the COVID‐19 pandemic could be connected to inflammatory and immune response mechanisms [239]. It is notable that fluoxetine has been demonstrated to markedly inhibit SARS‐CoV‐2 at a concentration of 0.8 µg/ml, leading to a decrease in the expression of viral proteins [240]. The research findings suggest that SSRIs, particularly fluoxetine hydrochloride and fluvoxamine maleate, are associated with a decrease in the severity of COVID‐19 [241]. The combination of itraconazole and fluoxetine constitutes a promising initial strategy for therapeutic interferences aimed at handling SARS‐CoV‐2 infection and alleviating the severe advancement of COVID‐19 [242].

Different types of antidepressants have the possibility to change the composition of the gut microbiota. Particularly, rumen cocci have been associated with the relief of depressive‐like behaviors [243]. Furthermore, it has been shown that medications like fluoxetine and amitriptyline can cause changes in both the gut microbiota and the functional dynamics of the gut microbiome in rat models [244]. Atypical antipsychotics, such as risperidone, have been linked to the emergence of drug‐induced metabolic syndrome, along with weight increase, obesity, and glucose intolerance [245]. Antipsychotic drugs have been linked to elevated circulating levels of leptin [246], which might play a role in the emergence of metabolic syndrome. This syndrome is marked by dysfunction in the operation of the pancreas, liver, adipose tissue, muscle, and endocrine system. Additionally, the bioaccumulation of gut bacteria could be a common mechanism through which drug efficacy and bacterial metabolism are altered. This occurrence has the capacity to affect microbiota composition, pharmacokinetics, adverse reactions, and individual drug responses [247].

#### 3.3.2 Limitations

Elevations in alanine aminotransferase levels exceeding three times the upper limit of normal were observed in patients receiving treatment with SSRIs, indicating the possibility for clinically significant drug‐induced liver injury (DILI) [248]. Furthermore, prolonged use of SSRIs may result in hyperpigmentation of the skin and nails [249], as well as alterations in platelet aggregation [250].

#### 3.3.3 Implications for patients

The administration of SSRIs and serotonin‐norepinephrine reuptake inhibitors is commonly linked to a variety of adverse effects. Commonly reported adverse effects include nausea, diarrhea, indigestion, gastrointestinal bleeding, and abdominal pain [251]. Additionally, these medications may contribute to weight gain and metabolic disorders [252], urinary retention [253], sexual dysfunction [254], hyponatremia [255], and other detrimental symptoms [256].

### 3.4 Drug‐Related Clinical Studies with Cross‐Organ Therapeutic Effects

We have compiled a table summarizing clinical studies related to the trans‐organ immune axis, covering studies in the liver–brain, liver–intestinal, liver–skin, brain–cardiac, brain–hepatic, brain–genital organ, and brain–intestinal axes. The table includes the type of study, study population, immune‐related pathways and targets involved, mechanism of action, interventions (drug and dosage), key findings and conclusions, and the focus of the study revolves around immune regulation, intestinal flora, metabolic pathways, and the effects of inflammatory factors on different organs. For example, rifaximin in the liver–brain axis improved HE, while GLP‐1 receptor agonists modulate metabolic function and reduce hepatic fat deposition in the liver–gut axis. In addition, the table mentions potential adverse effects of some drugs, such as irAEs with anti‐PD‐1 therapy and DILI with antidepressants. Overall, the table demonstrates the immune and metabolic modulatory effects of multiple drugs across different organs, providing clinical evidence in support of cross‐organ therapies (Table 1).

**TABLE 1:** Summary of immune‐targeted therapies across organ systems.

Organ–immune axis	Study type	Study name/reference	Study subjects (animal/Clinical)	Immune‐related pathways/Targets	Mechanism of action	Intervention (drug/dosage)	Key findings/conclusions	Clinical trial NCT number
Gut–brain	Clinical trial	Rifaximin‐a reduces gut‐derived inflammation and mucin degradation in cirrhosis and encephalopathy: RIFSYS randomised controlled trial [257]	38 patients with cirrhosis and hepatic encephalopathy (HE)	TNF‐α, IL‐17A, TLR‐4, gut microbiome	Reducing gut microbial translocation, decreasing mucin‐degrading bacteria, and inhibiting systemic inflammation induced by gut microbiota	Rifaximin‐a(550 mg BID)	Rifaximin‐a significantly improved HE severity, reduced infection risk, and enhanced neurocognitive function. It decreased Veillonella spp., Streptococcus spp., and improved gut barrier function.	NCT02019784
Gut–brain	Clinical trial	Modulation of the metabiome by rifaximin in patients with cirrhosis and minimal hepatic encephalopathy [258]	20 patients with cirrhosis and MHE	Endotoxemia, gut microbiome, fatty acids	Modulating gut microbiota and metabolic products to improve cognitive function and reduce endotoxemia	Rifaximin 550 mg BID, 8‐week treatment	Rifaximin significantly improved cognitive function, reduced endotoxemia, altered gut microbiota composition (e.g., decreased Veillonellaceae, increased Eubacteriaceae), and increased serum fatty acids.	NCT01069133
Gut–brain	Clinical trial	Rifaximin treatment in hepatic encephalopathy [259]	299 patients with cirrhosis and hepatic encephalopathy (HE)	Endotoxins, gut microbiota	Reduction of endotoxins and modulation of gut microbiota to lower HE recurrence	Rifaximin 550 mg twice daily for 6 months	Rifaximin significantly reduced the risk of HE recurrence and improved cognitive function. Compared with the placebo group, hospitalization rates in the treatment group decreased by 50%.	NCT00298038
Gut–brain	Clinical trial	Rifaximin is safe and well tolerated for long‐term maintenance of remission from overt hepatic encephalopathy [260]	392 patients with cirrhosis and hepatic encephalopathy (HE)	Endotoxins, gut microbiota	Improving gut microbiota composition and reducing endotoxin levels to prevent HE recurrence	Rifaximin 550 mg twice daily for ≥24 months	Long‐term use of rifaximin significantly reduced HE‐related hospitalization rates without increasing adverse events. Compared with the placebo group, the treatment group showed a significant reduction in hospitalization rates. No negative impact on survival was observed in HE patients.	NCT00686920
Gut–brain	Clinical trial	Randomized, double‐blind, placebo‐controlled study of a multispecies probiotic mixture in nonalcoholic fatty liver disease [261]	68 obese NAFLD patients (BMI ≥25 kg/m^2^, MRI‐PDFF liver fat ≥5%)	Gut microbiome, Inflammatory markers (TNF‐α, IL‐6)	Modulating gut microbiota to reduce systemic inflammation and hepatic fat accumulation	Probiotics (L. acidophilus, L. rhamnosus, L. paracasei, P. pentosaceus, B. lactis, B. breve), administered orally daily for 12 weeks.	The probiotic group showed a significant reduction in intrahepatic fat (IHF) (16.3% → 14.1%, *p* = 0.032); however, after adjusting for weight change, the reduction was no longer significant. Triglyceride levels significantly decreased (−34.0 mg/dL, *p* = 0.0033). No improvement in liver fibrosis was observed.	KCT0001588
Gut–brain	Clinical trial	Histological improvement of non‐alcoholic steatohepatitis with a prebiotic: a pilot clinical trial [262]	14 patients with biopsy‐confirmed NASH (NAS ≥5)	Gut microbiome (increased Bifidobacterium, decreased clostridium cluster I and XI), inflammatory factors (IL‐6, TNF‐α)	Modulating gut microbiota to reduce systemic inflammation, hepatic fat deposition, and inflammatory response	Oligofructose oral administration: 8 g/day for the first 12 weeks, 16 g/day for the next 24 weeks; control group received an isocaloric placebo (maltodextrin) for a total of 9 months.	The oligofructose group showed histological improvement, including a significant reduction in hepatic steatosis (*p* = 0.016) and a decreased NAS score. Bifidobacterium abundance significantly increased (*p* < 0.05), while Clostridium cluster I and XI significantly decreased (*p* < 0.05). IL‐6 and TNF‐α levels decreased, but the difference did not reach statistical significance due to the small sample size. No significant improvement in liver fibrosis was observed. Body weight and BMI remained unchanged between groups, indicating that improvements were independent of weight changes.	NCT03184376
Gut–brain	Clinical trial	A placebo‐controlled trial of subcutaneous semaglutide in nonalcoholic steatohepatitis [159]	Oligofructose oral administration: 8 g/day D24for the first 12 weeks, 16 g/day for the next 24 weeks; control group received an isocaloric placebo (maltodextrin) for a total of 9 months	GLP‐1 receptor pathway, metabolic regulation (insulin resistance), inflammatory markers (IL‐6, TNF‐α)	GLP‐1 receptor agonist improves insulin sensitivity, reduces weight, decreases hepatic fat deposition and inflammation to ameliorate NASH pathology.	Subcutaneous semaglutide (0.1 mg, 0.2 mg, 0.4 mg) daily for 72 weeks; control group received placebo	The remission rate of NASH (without fibrosis worsening) was 59% in the 0.4 mg group, significantly higher than in the placebo group (17%) (*p* < 0.001). Fibrosis improvement (≥1 stage, without NASH worsening) was observed in 43% of the 0.4 mg group and 33% of the placebo group (*p* = 0.48), with no statistical difference. Body weight reduction: The 0.4 mg group exhibited an average decrease of 13%, compared with only 1% in the placebo group. Key adverse events: nausea (42 vs. 11%), constipation (22 vs. 12%), and vomiting (15 vs. 2%), all of which were more frequent in the 0.4 mg group than in the placebo group. An increased risk of malignancies (breast cancer, endometrial adenocarcinoma, and T‐cell lymphoma) was observed, though no organ‐specific pattern was identified.	NCT02970942
Gut–brain	Clinical trial	Effect of dulaglutide on liver fat in patients with type 2 diabetes and NAFLD: randomised controlled trial (D‐LIFT trial) [263]	Sixty‐four patients with type 2 diabetes and concomitant NAFLD (LFC ≥6.0% as measured by MRI‐PDFF)	GLP‐1 receptor pathway, metabolic regulation (insulin resistance), and inflammatory markers (IL‐6, TNF‐α)	GLP‐1 receptor agonists improve the pathological features of NAFLD by enhancing insulin sensitivity, reducing body weight, and decreasing hepatic fat deposition and inflammation.	Participants received once‐weekly subcutaneous dulaglutide (0.75 mg for the first 4 weeks, followed by 1.5 mg) for a total of 24 weeks, while the control group received standard diabetes care.	Liver fat content (LFC) was significantly reduced in the dulaglutide group (from 17.9 to 12.0%, *p* < 0.0001), whereas no significant change was observed in the control group (from 17.1 to 14.8%, *p* = 0.058). GGT levels significantly decreased in the dulaglutide group (*p* = 0.025), while AST and ALT levels showed a downward trend but did not reach statistical significance. Body weight reduction: The dulaglutide group experienced an average weight loss of 4.3 kg, compared with 2.0 kg in the control group (*p* = 0.01). No significant improvement in liver fibrosis (LSM) was observed (*p* = 0.123). Dulaglutide was well tolerated, with mild gastrointestinal discomfort as the primary adverse event.	NCT03590626
Gut–brain	Clinical trial	Liraglutide safety and efficacy in patients with non‐alcoholic steatohepatitis (LEAN): a multicentre, double‐blind, randomised, placebo‐controlled phase 2 study [264]	Fifty‐two patients with biopsy‐confirmed NASH (BMI ≥25 kg/m^2^), including nine with concomitant type 2 diabetes	GLP‐1 receptor pathway, metabolic regulation (insulin resistance), and inflammatory markers (IL‐6, TNF‐α)	GLP‐1 receptor agonists improve the pathological features of NASH by enhancing insulin sensitivity, reducing body weight, and decreasing hepatic fat deposition and inflammation.	Patients received daily subcutaneous liraglutide (1.8 mg) for 48 weeks, while the control group received a placebo.	NASH remission rate (without fibrosis worsening): 39% (nine out of 23) in the liraglutide group, significantly higher than 9% (two out of 22) in the placebo group (*p* = 0.019). Fibrosis progression: Observed in 9% (two out of 23) of the liraglutide group, compared with 36% (eight out of 22) in the placebo group (*p* = 0.04). Body weight reduction: The liraglutide group experienced an average weight loss of 5.3 kg, compared with only 0.6 kg in the placebo group (*p* = 0.003). Liver enzyme levels: γ‐Glutamyl transferase (GGT) showed a significant reduction (*p* = 0.01), while ALT and AST remained unchanged. Key adverse events: Gastrointestinal discomfort was more frequent in the liraglutide group (81 vs. 65%), including nausea (46 vs. 38%) and reduced appetite (31 vs. 8%).	NCT01237119
Liver–immune	Clinical + animal study	Uncoupling immune trajectories of response and adverse events from anti‐PD‐1 immunotherapy in hepatocellular carcinoma [265]	32 HCC patients (Singapore), 29 HCC patients (Korea)	CXCR3^+^CD8^+^ T_EM_ cells (promoting immune‐related adverse events), TNFR1 and TNFR2	High TNFRSF1B (TNFR2) expression in tumor‐infiltrating Tregs; TNFR2 inhibition reduces immune‐related adverse events while maintaining ICB efficacy (TIL中Treg的TNFRSF1B).	Biweekly intraperitoneal injections of anti‐PD‐1 (RMP1‐14, 250 µg/mouse), anti‐TNFR1 (55R‐170, 250 µg/mouse), anti‐TNFR2 (TR75‐54.7, 500 µg/mouse)	TNFR2 inhibition preserves ICB efficacy while eliminating immune‐related adverse events.	NCT03695952
Brain–heart	Clinical trial	Effect of polymorphisms on the pharmacokinetics, pharmacodynamics and safety of sertraline in healthy volunteers [266]	46 healthy volunteers (24 males, 22 females)	Metabolic enzymes: CYP2C19, CYP2B6 (the main metabolic enzyme, gene polymorphisms affect the rate of drug metabolism). Transporter: ABCB1 (P‐glycoprotein, which affects drug distribution and clearance). Receptors: HTR2A, HTR2C (serotonin receptors, associated with efficacy and side effects)	Inhibits serotonin transporter (SLC6A4) and increases synaptic cleft serotonin concentration. Polymorphisms in CYP enzyme genes lead to metabolic differences that affect plasma concentrations (e.g., AUC increases in CYP2C19 intermediate metabolizers).	Sertraline 100 mg orally was given as a single dose to evaluate pharmacokinetics, pharmacodynamics and safety.	The effects of sertraline on the cardiovascular system are mainly manifested in a decrease in heart rate, gender and gene polymorphisms significantly regulate the risk of side effects, and women and patients with CYP2C19/CYP2B6 metabolism defects are more likely to have adverse reactions. The ABCB1 genotype may reduce the incidence of side effects by influencing drug distribution.	
Brain–liver	Clinical trial	Adverse event profiles of drug‐induced liver injury caused by antidepressant drugs: a disproportionality analysis [267]	Patients on antidepressant medications	5‐Serotonin (5‐HT) pathway	Inhibits 5‐HT reuptake and increases synaptic cleft 5‐HT concentration	Antidepressants (no specific dose mentioned)	Of the 324,588 cases of antidepressant use in the FAERS database, 10,355 were identified as DILI cases. Nefazodone, fluvoxamine, and clomipramine have the highest ROR for cholestatic injury. Mianserin, nefazodone, and maprotiline for hepatocellular injury. Nefazodone, clomipramine, and mirtazapine for the treatment of serious drug‐related liver disease. Only nefazodone causes liver failure signals. There was a significant association between DILI and nefazodone, with duloxetine and clomipramine associated with 3 DILI categories, with the exception of liver failure. Disproportionality analyses could not lead to a clear causal relationship between antidepressants and DILI, and more research is needed to assess the propensity of newer generations of antidepressants to cause DILI.	
Brain–reproductive organ	Clinical trial	Post‐finasteride syndrome and post‐SSRI sexual dysfunction: two clinical conditions apparently distant, but very close [268]	Males (patients treated with SSRIs for depression)	Neuroactive steroids (e.g., pregnenolone, progesterone, DHEA, testosterone, etc.); neurotransmitters (e.g., serotonin, dopamine, norepinephrine, etc.); gut microbiota	Inhibits 5α‐reductase and affects androgen metabolism. Affects the synthesis, release, and action of neurotransmitters. Alter the composition and function of the gut microbiota	SSRIs (e.g., citalopram, escitalopram, fluoxetine, fluvoxamine, paroxetine, sertraline, etc., no specific dose mentioned)	Treatment with finasteride and SSRIs may cause persistent sexual dysfunction that may persist even after discontinuation of the drug. These medications may cause sexual dysfunction and other adverse effects by affecting pathways such as neuroactive steroids, neurotransmitters, and the gut microbiota. PFS and PSSD may have similar pathophysiological mechanisms, involving the interaction of neuroactive steroids, neurotransmitters, and the gut microbiota.	
Brain–gut	Clinical trial	Improving medication tolerance: a pilot study in disorders of gut–brain interaction treated with tricyclic antidepressants [269]	Patients with disorder of gut–brain interaction (DGBI)	Serotonin (5‐HT) receptors; norepinephrine (NE) receptors; Dopamine (DA) receptors; signaling pathways related to the gut–brain axis	Negative expectations (nocebo effect) were alleviated through educational interventions, thereby improving tolerance to tricyclic antidepressants (TCAs). Affects the release and action of neurotransmitters and regulates the function of the gut–brain axis.	Tricyclic antidepressants (TCAs) (dose not specified)	A brief clinical intervention (about 30 s) can improve the patient's tolerance to TCAs. Patients in the enhanced information group reported a lower number of side effects and were significantly less bothersome with these side effects. Patients in the enhanced information group reported a higher proportion of symptom relief after 2 weeks of treatment.	

## 4 Perspectives of Cross‐Organ Immune Regulation

### 4.1 Evolving Insights into Systemic Immune Interactions

Knowledge pertaining to the organization of immune processes across multiple organs has dramatically been evolved in the era of postgenome science. The immune systems with regard to intestinal tract, liver system, and nerve cell system were assumed to act autonomously at the beginning of immunology, to regulate immune mechanisms within themselves. The latest research has revealed that these living organisms communicate with each other via a kind of ongoing back and forth in which an immunological event that takes place in one region of the intestine can trigger a measurable change in the immune scenery of the brain. This major shift in perspective has come because it was realized that there are complex biochemical messenging pathways that can drive coordinated activities between the immune system regions residing at disparate locations, that is, cellular processes that can mediate the immune system's communication over longer distances (over one cell layer), substantially changing our perception regarding the integrated immunological ecology of the whole body.

Recent studies validate that proinflammatory molecules emanating from the gastrointestinal tract–microbiota‐specific bioactive molecules play regulatory roles in liver immune function [270] and in brain immunoregulation [271]. Immune molecules from the liver has been shown to show the ability to globally prime the whole immune system beyond the local microenvironment within the liver [272]. Despite immunological role of the CNS being for a long time concealed behind the BBB and the blood‐retina barrier, the recent work outlines the significance of the CNS in systemic inflammatory response (including the so‐called neuro‐immune response cascades with system‐level effect) [273].

The traditional, organ‐specific approaches toward drug development are in the process of paradigm shift, as novel pharmacotherapies targeting specific organs seem to reveal, unexpectedly, trans‐organ immune modulatory functionality. This understanding of the interdependence of local and systemic immune communication fueled the design of multiorgan–immune‐modulating approaches exploiting anatomical crosstalk pathways toward therapeutic benefit.

These observations have stimulated a revolution in interrogating biological networks and are certainly reframing immunology's primary questions. Clearly, understanding intertissue signaling logic is immunology's new primary aim. Likewise, it is also becoming quite evident that designing novel informatics infrastructure to track the transmission of immune signals between different organs is less of an aspiration and much more of a requirement to explore uncharted areas to address multiorgan immunopathologies.

### 4.2 Exploring Techniques for Cross‐Organ Immune Regulation

Modern approaches like multiomics approaches, big data analysis, and organ‐on‐a‐chip modelling are key tools in understanding the immune system working in diverse organs, which will also be able to illuminate the key molecular mechanisms and guide the exploration for novel therapeutic strategies.

#### 4.2.1 Multiomics Approaches

With the advent of multiomics technologies (e.g., genomics, transcriptomics, proteomics, and metabolomics), the organ communication has been unraveled in‐depth. Among those, metabolomics seems most beneficial to characterize the gut–liver axis, as shown in Wang et al. [274] (using metabolomic analysis to identify the gut‐derived metabolites that significantly contribute to the liver dysfunction in regulating the pathogenesis of the NAFLD and liver fibrosis). In a series of their findings, the investigators highlighted the roles of products such as SCFAs and bile acids in such mechanisms and proposed them as potential targets in liver disease treatment [274]. In a different work, Zhang et al. [275] investigated how the bile acids regulate gut–liver communication, particularly in their role as mediators of driving liver inflammation and liver diseases.

Other than metabolomics, proteomics has been also used to further our understanding of immune regulation in organs. Guo et al. used proteomic analysis of the gut–brain axis and pinpointed gut microbiota impacted proteins in relation to the formation of neurodegenerative disease [276]. Similarly, Zundler et al. [277] showed how an integrative omics, consisting of transcriptomics, proteomics, and metabolomics, can offer further knowledge of the gut–liver axis. Such a multilevel framework allowed to identify key molecular circuits linking gut‐related inflammation to liver damage [277], while a recent targeted multiomics study investigated the routes along which gut microbiota affects host immunity and metabolism via cross‐organ networks [278]. With the help of spatial transcriptomics, single‐cell RNA sequencing and targeted bile acid metabolomics, researchers reported that absence of the microbiota resulted in abnormal B cell, myeloid cell, and T/NK cell distributions; bile acid circulation; and lipid metabolism balance. We conclude that multiomics alone will allow for a distinct appreciation of the host‐microbiota‐immune‐metabolic interactions in distinct organs and is poised to generate novel insights on new pathways targeting the microbiota–immune–metabolic cross‐talk [279].

#### 4.2.2 Big Data Analytics and Machine Learning

Big data analytics and machine learning are gaining relevancy as a means to manage the vastness of the data produced by multiomics studies. Such sophisticated tools highlight the bidirectional organ communication pathways, in particular those of a gut microbiota modulating cerebral functioning via an enteric‐neural pathway. Xu et al. [280]. applied big data methods to microbiome data sets that reported associations of specific microbial communities to the neuroinflammatory process in the brain They suggest that this result can direct potential cures for neurodegenerative disease. In an analogous work, Yan et al. [71] used machine learning techniques to model communications of the gut–brain–liver axis by using microbiota composition and metabolite measurements to infer disease progression. They argue this can be a useful approach toward personalized cures.

Kim and Sung [281] employed machine learning to analyze multiomics data regarding gut–liver axis, which identified important biomarkers associated with liver diseases and paved the way for predictive disease progression models. Within the same research field, in a second study they employed eight different machine learning algorithms to analyze the alcohol–gut microbiota–liver axis and early‐stage HCC detection with an outstanding AUC of 0.932. In this investigation, Guo et al. [282] identified key microbial players who mediated and moderated the role of alcohol in risk of HCC, thus demonstrating the power of machine learning to deconvolve the host–microbiota axis across organs. Guo et al. [276] also noted machine learning as an approach to uncover associations between gut microbes and brain function, in the specific example of microbial signatures associated with cognitive decline.

#### 4.2.3 Organ‐on‐a‐Chip Models

Organ‐on‐a‐chip has been considered as an alternative in vitro platform for testing organ–organ communication in a confined environment. Microfluidic devices are capable of simulating physiological conditions of human organs and facilitates interorgan observation in real‐time. Kim et al. [283] established gut–brain chip to identify how microbial metabolites trigger neuroinflammations. They identified some metabolites that could either exacerbate or minimize inflammation, thus giving possible targets for neurological disease treatments [283]. Another study by Bauer et al. [284] employed the gut–liver chip, and studied how signals sent by the gut, can influence the function of the liver particularly during liver fibrosis. Their results were able to shed some light on the intricate interaction between the gut and liver [284].

Zhang et al. [285] used a multiorgan chip model to study the gut–liver–brain axis, showing that inflammation starting in the gut can affect both liver and brain function—contributing to conditions like AD and liver cirrhosis. Recently, the development of the gut‐on‐a‐chip (GOC) systems has remarkably enhanced our capability to simulate gastrointestinal operations. Such microfluidic systems offer a better representation of the dynamic gut environment and thus are believed to be more appropriate tools to model drug absorption and metabolism than static cultures or animal models. GOC systems were found particularly valuable in the field of drug development: they were used to evaluate the oral drug bioavailability as well as to model a disease state. GOCs can be combined with other organ chips to model multiorgan interactions in order to better predict behaviors of drugs throughout systems and systemic toxicities [286]. In addition to drug testing, GOCs now permit the coculture of synthetic gut microbiomes to conduct thorough research into how dietary intake impacts gut microbiomes. Such models enable testing of microbiota interactions, probiotic activity, and control of pathogenic bacteria in a physiologically relevant gut‐like environment. By integrating bacterial consortia with varied nutrients, researchers gain new insights into how diet shapes microbiota composition and metabolism—advancing strategies for managing gut health through nutrition [287]. Furthermore, developments in organ‐on‐a‐chip techniques have facilitated to construct multiorgan systems that enable to get a better grasp on how multiorgan communication can be involved in the development of disease [277].

## 5 Conclusion

The immune connections between organs are complex and profound, playing a key role in the progression of diseases such as inflammation and cancer. In recent years, cross‐organ immune regulation has become a research hotspot. Numerous pieces of evidence indicate that the interaction between organs is of great significance for maintaining the overall balance of the body and determining the outcome of diseases. An in‐depth exploration of these organ axes can reveal how the immune response of one organ widely affects the pathophysiological processes of another organ, and analyze how the immune response overcomes spatial barriers to achieve precise regulation between distal tissues, thereby triggering protective physiological effects or pathological changes. A comprehensive analysis of these complex interaction mechanisms will help develop more comprehensive and integrated treatment strategies, which is expected to improve treatment outcomes and minimize adverse reactions to the greatest extent.

In conclusion, driven by technological progress, the field of cross‐organ immunomodulation is undergoing significant evolution. This helps to clarify more thoroughly the complex network of immune communication among various organs, and the prospects for developing comprehensive treatment strategies are becoming increasingly optimistic. Looking ahead, a deeper understanding of cross‐organ immune regulation mechanisms is crucial for addressing the systemic characteristics of many diseases and improving patient prognosis through more personalized and comprehensive treatment approaches.

## Author Contributions

Jie Dou, Jinzuo Jiang, and Yangtao Xue were responsible for drafting the main manuscript. Xiaoqi Jiang and Yongzhuo Jiang participated in the writing and revision of the manuscript. Yangtao Xue, Xiaoqi Jiang, and Jinzuo Jiang were responsible for drawing the diagrams. Junjie Xu provided revision suggestions for the manuscript and, together with Peng Xiao, oversaw the writing process. All authors read and approved the final manuscript.

## Ethics Statement

The authors have nothing to report.

## Conflicts of Interest

The authors declare no conflicts of interest.

## Data Availability

All data used to support the findings of this study are included within the article.
